# The interplay between ecological networks drives host-plasmid community dynamics

**DOI:** 10.1371/journal.pcbi.1014339

**Published:** 2026-05-26

**Authors:** Ying-Jie Wang, Kaitlin A. Schaal, Johannes Nauta, Armun Liaghat, Manlio De Domenico, James P. J. Hall, Shai Pilosof

**Affiliations:** 1 Department of Life Sciences, Ben-Gurion University of the Negev, Beer-Sheva, Israel; 2 Department of Evolution, Ecology and Behaviour, Institute of Infection, Veterinary and Ecological Sciences, University of Liverpool, Liverpool, United Kingdom; 3 Department of Physics and Astronomy “Galileo Galilei”, University of Padova, Padova, Italy; 4 Department of Ecology and Evolution, University of Chicago, Chicago, Illinois, United States of America; 5 Padua Center for Network Medicine, University of Padova, Padova, Italy; Abdus Salam International Centre for Theoretical Physics, ITALY

## Abstract

Plasmids drive evolution by transferring traits across microbial hosts. Transmission depends on both host–plasmid (infection) and plasmid–plasmid (compatibility) interactions, yet how the structure of these networks shapes transmission remains poorly understood. We hypothesized that these two ecological networks interact in non-additive ways to influence community outcomes. To test this, we developed a stochastic agent-based model that embeds both network structures and simulates coupled host–plasmid dynamics. We systematically varied the structure of each network, both individually and in combination, to isolate the effect of structure on host-plasmid dynamics. A modular (interactions organized into clusters) and hub (interactions concentrated on the highly connected) plasmid-plasmid compatibility network promoted transient host coexistence, while a modular host-plasmid infection network promoted plasmid diversity and stable host coexistence. Importantly, structured networks interacted non-additively, and their impact was most apparent when plasmid carriage imposed a moderate fitness cost on hosts. For example, combining a modular infection network with a hub compatibility network reversed the expected plasmid prevalence patterns, demonstrating that the structure of one network can counteract the effects of the other. We further re-parameterized our model to recapitulate empirical host-plasmid community dynamics, showing that infection network structure can strongly shape plasmid prevalence even in the presence of substantial biological heterogeneity. Our results highlight the necessity of jointly considering host–plasmid infection and plasmid–plasmid compatibility networks to understand host–plasmid community dynamics and their eco-evolutionary potential. More broadly, this work provides an initial mechanistic framework for generating testable hypotheses and underscores that systems involving multiple hosts and infectious agents require explicit consideration of how different ecological networks interact to shape community dynamics.

## Author summary

Plasmids are genetic elements that can move between bacteria and help them adapt and evolve by horizontally transferring traits such as metabolism or antibiotic resistance. Plasmid spread in microbiomes is shaped by interactions with bacterial hosts and other plasmids. However, how the combined structure of host–plasmid and plasmid–plasmid interactions influences microbial community dynamics remains unclear. We explored this fundamental question using a computational model and an experiment, to understand how two ecological networks interact: one network describing which plasmids infect which bacteria, and another describing which plasmids coexist with other plasmids inside bacteria. Our results demonstrate that understanding the dynamics of bacteria, plasmids, and other mobile genetic elements requires considering the interplay between multiple types of ecological networks. Our work provides a generalizable framework for understanding how diverse ecological interactions jointly shape community dynamics, directly addressing fundamental questions at the intersection of community ecology, network theory, and infectious disease dynamics.

## Introduction

Plasmids play a vital role in the eco-evolutionary dynamics of host (bacteria) communities. As extrachromosomal mobile genetic elements (MGEs), plasmids can spread among host organisms, accelerating adaptation by transferring genes that confer advantageous traits such as antibiotic resistance [1–3]. Plasmids can also impose fitness costs on their hosts, which emerge from conflicts between chromosomal- and plasmid-encoded genes [4,5]. However, these cost-benefit interactions between plasmids and their hosts are shaped by other microbiome interactions, such as plasmid co-infection and host competition, which together influence plasmid dynamics and the coexistence of their constituent hosts [6–12]. The impact of this interplay is often overlooked in studies of host-plasmid community dynamics.

Plasmids can interact with multiple hosts as they transfer across populations, creating patterns of host infection shaped by variation in within- and between-population conjugation rates, compatibility with host genetic backgrounds, and host anti-plasmid defense systems [13–15]. Plasmids can also compete with one another for hosts, using mechanisms such as (in)compatibility groups, toxin-antitoxin systems, and plasmid-encoded defense systems to exclude one another [16–22]. While some studies acknowledge the importance of multiple interaction types, they focus on simplified systems, such as multihost-uniplasmid [7] or unihost-biplasmid systems [8]. However, in nature, hosts and plasmids form complex structures of interactions [23]. The few studies which consider multihost-multiplasmid systems omit or understate plasmid co-infection [24–26]. As a result, little is known about how the interplay of multiple interaction structures, specifically between host-plasmid interactions and plasmid co-infection, shapes community dynamics and stability. This gap hinders our ability to predict the assembly and evolutionary trajectories of these communities, particularly their potential in genetic innovation and adaptation. Nevertheless, addressing it is challenging because it requires connecting multiple interaction types in complex communities to dynamics—which is hard to do experimentally.

The interplay between different interaction types can be studied using ecological networks, which encode interactions (links) between multiple hosts and plasmids (nodes). Ecological networks are valuable tools for studying how network structure (i.e., the pattern in which interactions are distributed across species) affects community dynamics and species coexistence [27,28]. Considering multiple ecological networks allows us to study how distinct, interconnected networks affect community dynamics [12,29–31]. However, there is a paucity in studies that explicitly link dynamics to the interplay between networks, leading to a gap in our understanding of structure-dynamics in complex ecological communities.

**Fig 1:**
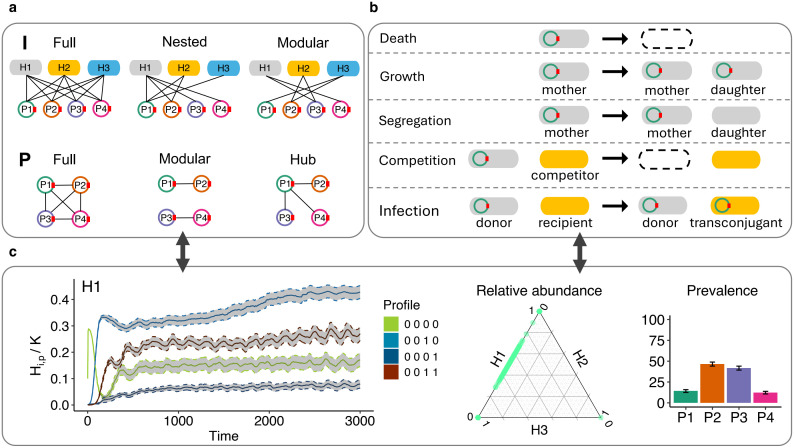
Model overview and experimental design. **(a)** The structures of the bacterial host-plasmid infection network **I** (which plasmid infects which hosts; top row) and plasmid-plasmid compatibility network **P** (which plasmids are compatible with each other and can therefore co-reside in a host individual; bottom row). H1, H2, H3 are the three hosts and P1, P2, P3, P4 are the four plasmids. The ‘full’ structures serve as controls. We used a factorial experimental design of the nine structure combinations. **(b)** Illustration of the five main events in the model (see Methods for detailed model explanations). Dashed ovals indicate deaths of individual cells. **(c)** An example of results of infection dynamics for the case of a modular **I** and full **P**, including subpopulation dynamics (left), final relative abundance of host populations of all replicates (middle), and the mean and SE of final plasmid prevalence across replicates at the end (t = 20000 hours) of the simulation (right).

Our goals were to (1) test how variation in the structures of two fundamental interaction networks: the host–plasmid infection network and the plasmid–plasmid compatibility network—jointly shape host-plasmid dynamics, and (2) determine whether the joint effects of infection and compatibility networks can be inferred from either network in isolation. While other interaction types exist (e.g., host-host interactions such as competition [7,32] and horizontal gene transfer (HGT) [24,33]), we focus on infection (a host-plasmid interaction) and plasmid compatibility (a plasmid-plasmid-interaction, Fig 1a) because these interactions are significantly understudied in a network context despite being fundamental to host-plasmid communities.

Theory and empirical observations suggest that infection and compatibility networks regulate distinct but interconnected processes: infection structure governs which hosts plasmids can colonize, whereas compatibility structure dictates whether plasmids can coexist within hosts. Co-infection can, in turn, modify host growth, plasmid persistence, and opportunities for horizontal transfer [8,9,17]. Infection structure shapes the sequence and frequency with which plasmids encounter one another within hosts, while compatibility structure feeds back on infection success by altering plasmid persistence, competitive outcomes, and host-level costs. Consequently, the effect of a given structure in one network depends on the structure of the other, such that identical infection networks can give rise to different community-level dynamics under different compatibility architectures, and vice versa. This coupling provides a natural mechanism for non-additive effects, whereby combined outcomes cannot be inferred from either network in isolation. We therefore hypothesize that these networks jointly constrain the set of host subpopulations that plasmids can occupy, but do so in non-independent ways.

To test our hypothesis, we developed a stochastic, agent-based model that incorporates the structure of both networks and simulates the dynamics of host and plasmid populations (Fig 1). We systematically varied the structure of each of these networks, either individually or simultaneously, to isolate their independent and combined effects on community dynamics. Our model omits accessory genes that provide plasmids with advantages (e.g., antibiotic resistance), so that network structures can be compared within a homogeneous abiotic environment. While the model is broadly applicable to a wide range of hosts and infectious agents, we focus on bacteria and plasmids throughout.

## Results

### Network definitions

We first define the two networks used in our study. The host–plasmid infection network (**I**) is a bipartite network whose nodes consist of hosts and plasmids, and whose links indicate that a given plasmid can infect a given host. The plasmid–plasmid compatibility network (**P**) is a unipartite network whose nodes are plasmids, and whose links indicate that two plasmids are able to co-reside within the same host. To understand the relationship between network structure and dynamics in detail and from core principles, we intentionally used small networks (Fig 1a). While this choice was primarily conceptual, focusing on tractability and interpretability, it also aligned with practical considerations, as simulating larger networks becomes computationally intensive (see model limitations in materials and methods). Multiple biological processes can mediate plasmid infection, such as failure of transfer, long-term fitness costs, replication constraints, or incompatibility [23]. Below, we introduce the mechanisms behind some relevant structures. Note that we chose to focus on ‘non-infection’ as the relevant phenotype of interest, as this is what the infection network encodes, whilst remaining agnostic about the biological processes involved. We focused on the effects of the end structures (i.e., effective non-infection and incompatibility), because this is what the infection and compatibility networks encode.

### Network structures

#### Infection network structures.

Previous studies of host-MGE infection networks have generally focused on phages, while the properties of multihost-multiplasmid infection networks remain poorly studied owing to technical difficulties in connecting plasmids with hosts (see [26,34] for examples) [23]. Nevertheless, given that plasmids are also infectious MGEs, a similar theory can be used to represent the structure of host-plasmid interactions.

Host-phage networks often exhibit modular and nested structures [12,35,36] (e.g., Fig 1a, top row). A modular network is partitioned into clusters (modules) of microbes and MGEs that interact densely with each other but sparsely with those outside the module. Nestedness reflects instead patterns of specialization where specialist microbes are infected by subsets of MGEs that in turn also infect the more generalist microbes [35]. These two structures are ultimately signatures of host-MGE coevolution and emerge from the co-evolutionary arms-race typical to infectious systems [12,37,38]. For example, bacteria–virus networks evolve nestedness under directional arms race dynamics, but not under fluctuating selection [39].

Modular infection networks can arise from a phylogenetic HGT barrier, where HGT occurs more often between closely related species [40,41]. Theory shows that modularity can also emerge from the evolution of niches for phage growth, whereby bacteria are resources that a group of phages is able to infect because it has overcome bacterial defenses [12]. In plasmids, factors that affect HGT and immunity also exist and include plasmid mobility genes, replication genes, and CRISPR defense systems [23,42–44]). This observation that modules reflect infection niches is supported by a study showing that antibiotic resistance (ABR) genes carried on plasmids would generate a more generalized infection pattern [34].

Nestedness indicates a hierarchy of infection patterns, which itself can reflect resistance hierarchy among hosts and infection ability among MGEs [36]. A nested structure can result from a sequence of adaptations whereby new bacterial mutations confer bacterial resistance to recently evolved phages while maintaining resistance to past phages. This is consistent with arms race coevolutionary dynamics, where hosts and phages evolve to increase their range of resistance [37–39]. A nested infection network can also arise from a trade-off between infecting many hosts and adapting to each host [45].

A rare multihost-multiplasmid study that has provided a census of plasmid-bacteria interactions in an infection network context [26] found that when putative ABR plasmids are excluded from the network, the network was more modular and less nested, because putative plasmids carrying ABR genes are advantageous and therefore, more widespread, connecting different parts of the network. In our study, we do not consider beneficial plasmid traits, which provides further support for choosing both modular and nested structures.

Given that modularity and nestedness are the dominant structures in host-MGE networks, and the empirical and theoretical evidence detailed above, we defined three structures for **I**: (1) ‘Full’, wherein all hosts can host all plasmids. This structure served as a reference (control). (2) ‘Nested’, wherein specialist plasmids interact with a subset of hosts with which more generalist plasmids interact [35,36,38,39]. (3) ‘Modular’, wherein plasmid-host interactions form three distinct modules: two peripheral modules of hosts that are infected by distinct subsets of plasmids, and a bridge module where hosts can host a subset of plasmids from each of the peripheral modules (Fig 1a, top row). Host H2 and plasmids P2 and P3 in the bridge module are termed the bridge host and bridge plasmids.

#### Plasmids compatibility network structures.

Unlike infection networks, MGE co-infection networks are poorly studied. Although there are studies of plasmid co-infection [17,46], no study has explicitly considered co-infection networks or analyzed their structures. We therefore suggest two plausible structures based on well-described molecular mechanisms. An important feature of plasmids is the immunity (e.g., CRISPR IV) and compatibility mechanisms they carry, which have a strong impact on the ability of plasmids to co-reside in a host cell due to shared replication or partitioning mechanisms [16,18,20–22,47]. These molecular mechanisms can generate a modular structure via immunity or incompatibility group-mediated plasmid-plasmid interactions that impede a stable coexistence within the same host, as was also theoretically shown in phage-bacteria systems [12].

Another important feature influencing plasmid compatibility is the presence of genes that reduce conflict or promote functional complementarity. For instance, plasmids that carry alternative replication or segregation systems that minimize interference with co-resident plasmids, or genes that encode functions complementing those of co-resident plasmids, are more likely to coexist with a broad range of plasmid types [8]. Such a feature can give rise to a ‘hub’ structure, in which the hub plasmids are rare among plasmids but disproportionately prevalent (i.e., widespread in the communities), as they can co-reside with many others. Novel incompatibility groups would encounter little direct conflict and thus be able to coexist broadly. This scenario is plausible because even small molecular changes can generate coexistence between otherwise identical plasmids. For example, a single–base pair mutation in the replication machinery was shown to produce a plasmid variant compatible with its own ancestor, effectively creating a new compatibility type [48].

Given the potential relevance of modular and hub structures in plasmid-plasmid networks, and the theoretical mechanisms detailed above, we defined three structures for **P**: (1) ‘Full’, wherein all plasmids are compatible, serving as a reference (control). (2) ‘Modular’, wherein plasmids in the module are compatible with each other. (3) ‘Hub’, wherein a sole hub plasmid is the only one compatible with others, while non-hub plasmids have few interactions and can co-reside with only a limited set of plasmids.

#### The model links host-plasmid dynamics with network structures.

To understand how network structures affect host-plasmid dynamics, we developed an agent-based model that incorporates five demographic and stochastic events: growth, death, plasmid loss by segregation, competition between hosts, and plasmid transmission by conjugation (Fig 1b). We employed a 3 × 3 factorial experimental design of the **I** and **P** structures. In each experiment we tracked the quantity *H*_*i*,*p*_, which is the abundance of a microbial host population *i* that can be infected with a combination of plasmids, forming subpopulations with a plasmid profile *p* (Fig 1c). We included three hosts (H1-3) and four plasmids (P1-4). We used a binary notation system to describe the plasmid profile of each host subpopulation. For example, the total population abundance $H_{i}=\sum_{p}H_{i,p}$ of a host *i* might be split into two subpopulations 0000 and 0011. The first subpopulation is plasmid-free and the second is infected with plasmids P3 and P4.

To focus on the effects of network structure, we assumed that all plasmids and host populations have identical traits (e.g., growth and death rates, conjugation rates). That is, host and plasmid types exist in the same trait niche, differing only in their network niche (node position in the interaction networks). We modeled host competition for a shared limiting resource by applying a community-wide carrying capacity [49]. This formulation preserves the interdependence between plasmid costs and competitive pressure, which necessarily unfold across hosts drawing on the same resource pool. Assigning each host a separate carrying capacity would artificially decouple these biological feedbacks.

We assume that plasmids are costly, and do not consider the possible effects of beneficial accessory genes (e.g., antibiotic resistance). This allows us to isolate the network effects on plasmid persistence and community dynamics from the effects of positive selection for plasmid-encoded traits. Understanding plasmid maintenance in the absence of such selection is a necessary first step for assessing how network structure shapes community outcomes. The insights we obtain extend broadly to infectious agents that typically do not confer benefits to their hosts. Moreover, we applied a sufficiently high conjugation rate for infection, and a multiplicative cost for plasmid co-infection (representing a slight positive epistasis). Also, we assumed the host dynamics to be faster than infection dynamics, such that host dynamics go to equilibrium before infection dynamics do. Doing so allows us to focus very specifically on the consequences of the network structure.

For each experiment, we calculated: (1) relative host abundance: the relative abundance of each host population out of the total community abundance; (2) plasmid prevalence: the fraction of host individuals across all populations which are infected with the plasmid; (3) host population composition: the proportions of subpopulations, each defined by a plasmid profile, within the host population; (4) host coexistence probability: the fraction of simulations in which hosts coexisted at any given time point. We distinguish between transient coexistence, in which hosts coexist for a certain period of time (thus, a delayed extinction indicates a longer transient coexistence), and stable coexistence, in which by the end of the simulation two or three hosts have reached non-zero equilibrium densities.

### Structured plasmid compatibility networks promote transient host coexistence

**Fig 2:**
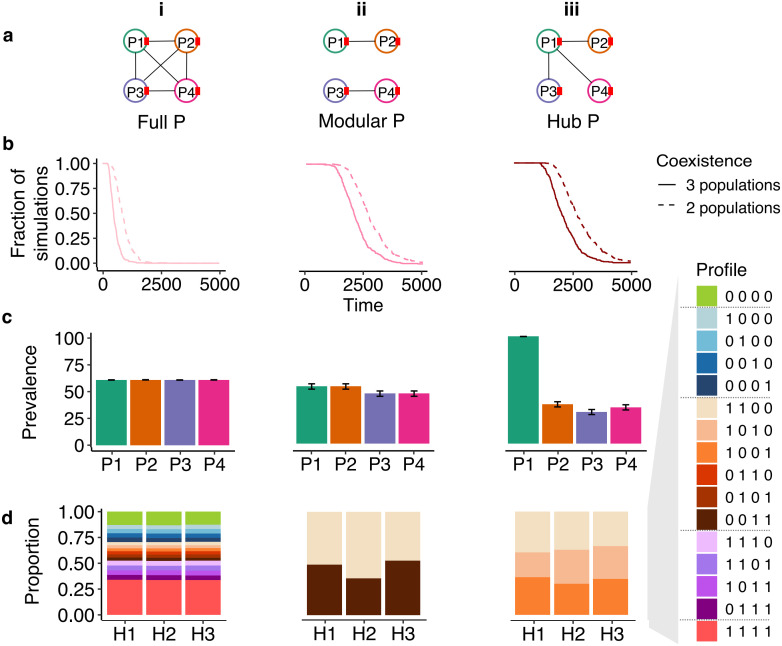
The effect of structure in the plasmid compatibility network P under a full infection network I. Columns i, ii, and iii represent different **P** structures. **(a)** Illustration of the different structures. **(b)** The dynamics of coexistence probability (fraction of simulations) of host populations. **(c)** Mean and SE of final plasmid prevalence across all replicates. **(d)** Final host population composition, averaged across replicates in which that host population survived (only populations surviving in >5 replicates were considered). Profiles represent the host subpopulations (e.g., the profile 1000 represents a subpopulation hosting only P1).

To understand the impact of the plasmid compatibility network **P** on community dynamics, we compared community dynamics under various structures of **P** while retaining a full infection network **I** (Fig 2a). Under full **I** and full **P** (a control treatment), only one host population survived (Fig 2b(i); Fig A in S1 Appendix). As expected, the identity of the host varied stochastically across replicate simulations, and the overall probability of a specific host surviving was about 1/3, because none of the hosts has an intrinsic advantage. Throughout the simulation, the probability of host transient coexistence rapidly decreased to zero (Fig 2b(i)). The host transient coexistence pattern was driven by neutral demographic stochasticity that eventually caused extinction, as well as by the high proportion of subpopulations infected by all four plasmids that induced higher costs and sped up extinction (Fig 2d(i); Fig B in S1 Appendix). These heavily infected subpopulations acted as the main plasmid donors, continually re-infecting available hosts. At the final time point, all subpopulations (i.e., all possible plasmid profiles) of the surviving host were still present, and population composition was the same regardless of which specific host survived (Fig 2d(i)). The four plasmids reached the same prevalence, infecting slightly more than half of the individuals of the sole surviving host (Fig 2c(i)). Although the plasmid prevalence at the end of the simulation can be analytically derived, the subpopulation composition depends on the infection rate (S1 Appendix).

Introducing structure to plasmid compatibility drastically affected host transient coexistence patterns. When **P** had a modular or hub structure, a random host population survived while the other two went extinct by the end of the simulation due to demographic stochasticity, but the process took up to 3 times longer (Fig 2b), reflecting an increased potential of transient coexistence. The average prevalence of the plasmids was similar to each other under each **P** structure, except for P1 infecting 100% of its hosts as the hub plasmid (Fig 2c(ii-iii)). To explain the prolonged host coexistence and the plasmid prevalence patterns we compared the final population compositions to those resulting from the full **P** experiment (Fig 2d). Although positive epistasis was implemented via multiplicative costs under co-infection (see equation 2 Methods), infections with four plasmids were still highly costly, accelerating host extinction. When **P** is structured, no host subpopulation can be infected with all four plasmids. Instead, the surviving host population in each replicate simulation was comprised solely of subpopulations infected by two plasmids (either the two plasmids that shared a module, or the hub plasmid and one other; Fig 2d(ii-iii)). Here too we can analytically derive the plasmid prevalence at the end of the simulation but the subpopulation composition depends on the infection rate (S1 Appendix).

Structure in the plasmid compatibility network **P** could therefore alter host dynamics in nature through plasmid cost and patterns of conjugation. Moreover, while all of the communities ultimately collapsed to a single host population over the course of the simulations owing to the strict competition between hosts, **P** structures qualitatively altered plasmid fates and prolonged the period of host coexistence. In a natural community, these dynamic outcomes would provide greater opportunities for host and plasmid evolution.

### A modular infection network promotes stable host coexistence and plasmid diversity

**Fig 3:**
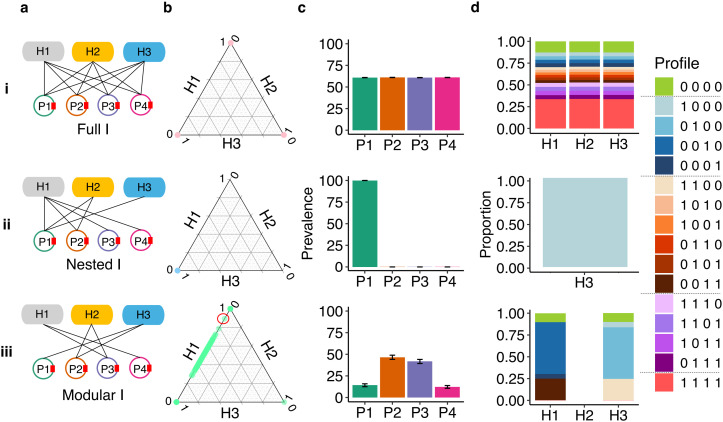
The effect of structure in the infection network I under a full plasmid compatibility network P. Rows i, ii, and iii represent different **I** structures. **(a)** Illustration of the structures of **I**. **(b)** Final relative abundance of host populations. Each dot represents a replicate. Dots on the vertices had no coexistence (i.e., only one population survived), while dots on the edges had coexistence of two populations. For example, the green dot marked with a red circle in b(iii) represents a community with relative abundances of 0.9 for H1, 0.1 for H3, and 0.0 for H2. **(c)** Mean and SE of final plasmid prevalence across all replicates. **(d)** Final host population composition, averaged across replicates in which that host population survived (only populations surviving in >5 replicates were considered). Profiles represent the host subpopulations (e.g., the profile 1000 represents a subpopulation hosting P1). The green dot at H2 = 1 in b(iii) is a rare outcome out of 300 simulations.

Next, we investigated the impact of infection network **I** (Fig 3a) on community dynamics. While under full **I**, each of the three host populations had an equal chance to out-compete the others, under nested **I** (hierarchical infections) the specialist low-degree host H3 consistently excluded the other two hosts (Fig 3b (i-ii)). As H3 could only be infected by the generalist plasmid P1, the other plasmids were lost from the community (Fig 3c(ii)). This outcome resulted from the lower net fitness cost to H3 of being susceptible to only one plasmid (Fig 3d(ii); Fig C in S1 Appendix). Notably, this is under the model’s assumption that all plasmids have the same cost to their hosts; more hosts and plasmids might survive if the generalist plasmid P1 has a much higher cost to H3 than other hosts. A nested infection network is therefore unlikely to be maintained without influence from host-specific plasmid cost or external factors (see Discussion).

In contrast, a modular **I** was the only structure that enabled stable host coexistence. The peripheral hosts H1 and H3 were both present at the end of the simulation (coexistence probability of ≈0.4), while the bridge host H2 went extinct (coexistence probability ≈0; Fig 3b(iii); Figs D-F in S1 Appendix). The rapid extinction of H2 was due to quick co-infection by P2 and P3, which resulted in a higher fitness cost compared to H1 and H3 (Figs G-I in S1 Appendix). The bridge plasmids P2 and P3 had higher final prevalence than the peripheral plasmids P1 and P4 (Fig 3c(iii); S1 Appendix). P2 and P3 maintained higher prevalence throughout the simulation because earlier in the simulation when H2 was still present, it acted as a source and increased the rate at which H1 and H3 were infected by these bridge plasmids (Fig J in S1 Appendix). The longer-term success of P2 and P3 after their source H2 became extinct is an example of the long-term impact on communities caused by a transient, though unsuccessful population [50].

### Combined structures generate non-additive dynamics

Our results so far show that modular networks (especially the infection network) promote the maintenance of diversity. Specifically, we observed prolonged host coexistence with modular **P**, and stable host coexistence with modular **I**. Communities with such structures may therefore be more likely to produce evolutionary innovation. However, we have only explored structured **I** or **P** separately, while maintaining the other as a fully-connected control. In natural communities, structures likely exist in both of these networks simultaneously, so we next explored the interplay between the two.

**Fig 4:**
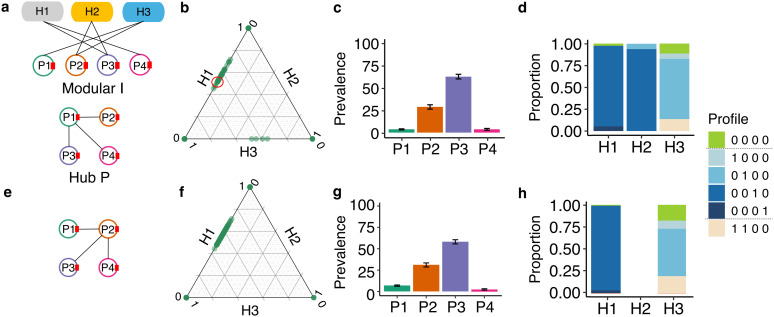
The joint effect of structured networks: modular I and hub P. **(a)** The modular network structure of infection **I** and the hub network structure of plasmid compatibility **P** with P1 as the hub plasmid. **(b)** Final relative abundance of host populations. Each dot represents a replicate. Dots on the vertices had no coexistence (i.e., only one population survives), while dots on the edges had the coexistence of two populations. For example, the green dot marked with a red circle represents a community with relative abundances of 0.5 for H1, 0.5 for H3, and 0.0 for H2. **(c)** Mean and SE of final plasmid prevalence across all replicates. **(d)** Final host population composition, averaged across replicates in which that host population survived (only populations surviving in >5 replicates were considered). **(e)** Choosing an alternative hub **P** (P2, in the bridge module of the infection network, with a broader host range than P1) resulted in similar **(f)** final relative host abundance, **(g)** plasmid prevalence, and **(h)** host composition. Profiles represent the host subpopulations (e.g., the profile 1000 represents a subpopulation hosting P1).

Due to the system’s non-linearity, it is not straightforward to anticipate how combinations of structures in both networks will interact, or whether their joint effects can be inferred from each network in isolation. Yet we did find a clear case from all the combinations where the two structures counteracted each other’s expected influence. Under a modular **I** combined with a hub **P**, the hub plasmid (i.e., the plasmid compatible with all others) did not become the most prevalent, contrary to expectation (Fig 4a; see Figs A, D, and J in S1 Appendix for additional combinations). Under a fully connected **I**, the hub plasmid P1 indeed reached fixation in hosts because all hosts became co-infected with P1 (Fig 2c(iii)). However, when **I** was modular, P1 ended with very low average prevalence, whereas P3 reached the highest average prevalence (Fig 4c).

These patterns resulted from the interaction between the structures of **I** and **P**: although the hub plasmid P1 was compatible with the other three plasmids, the peripheral host H3 was its only possible host (Fig 4a). H3 could therefore be co-infected by P1 and P2. Under a full **P**, the other peripheral host H1 could also be co-infected, but under a hub **P** this was no longer possible. This structure combination led to a higher net fitness of H1 compared to H3 (Fig 4b, d; Fig I in S1 Appendix). In addition, while under full **P** H2 always went extinct (Fig 3biii), under structured **P** it coexisted with host H3 in some simulations (Fig 4b). This is because under structured **P** H2 was not susceptible to the burden of co-infection (Fig I in S1 Appendix). Importantly, those patterns remain consistent regardless of the choice of the hub plasmid (Fig 4e-h), but due to a different mechanism (S1 Appendix). Overall, these results suggest that the infection network can counteract the effects of the plasmid compatibility network, especially when both are structured.

### Plasmid cost interacts with network structure

Until now, we have assumed a moderate plasmid cost ($c_{\alpha }=0.3$), corresponding to a 30% reduction in host growth rate relative to plasmid-free hosts. This choice reflects empirical estimates and acknowledges that plasmid cost directly affects host fitness, potentially leading to the exclusion and extinction of particular host populations [17,51–53]. Because compatibility determines whether plasmids can co-reside, it can further amplify or mitigate these fitness effects through their aggregate (here, multiplicative) impact on host growth. Network structure therefore shapes the distribution of fitness differences among hosts, influencing coexistence and associated community-level patterns. We hypothesize that plasmid cost modulates the influence of network structure on community dynamics. When plasmid carriage is moderately costly, growth penalties should interact with host position in the infection network, amplifying or dampening structural differences and limiting how far plasmids can spread through the host community [34]. In contrast, when plasmid costs are negligible or extreme, fitness effects should dominate dynamics, reducing the influence of network structure and weakening its impact on host coexistence and plasmid prevalence.

**Fig 5:**
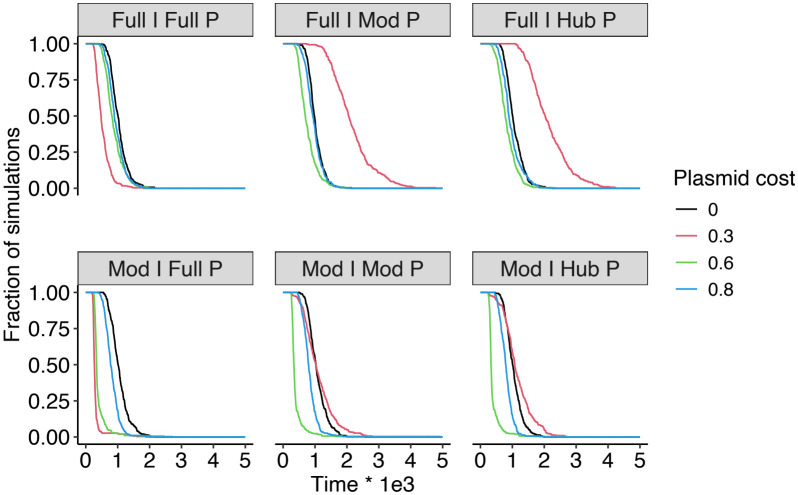
Dynamics of microbe coexistence of three populations across plasmid costs under full infection structures (top row) and modular infection structures (bottom row). The dynamics of coexistence probability (fraction of simulations) is calculated as the proportion of replicates with complete(3-population) coexistence out of total number of replicates across time. Time was only plotted to t = 5000 where most probabilities had dropped to zero.

To test this hypothesis, we systematically varied plasmid cost in our simulations and examined how its interaction with network structure shaped host coexistence, plasmid prevalence, and the distribution of plasmid-free subpopulations. As predicted, the absence of plasmid cost or when plasmid cost is high ($c_{\alpha }>0.3$) diminishes the effects of network structures on host coexistence. Specifically, when the compatibility network is structured, hosts coexist for longer under moderate cost compared to no cost or high cost, and this effect is stronger when the infection network is modular (Fig 5, Fig K in S1 Appendix), and homogenizes the host survival probability at ca. 1/3 (Fig L in S1 Appendix). The absence of plasmid cost reduces the impact of the network on hosts by reducing the burden associated with being exposed to a plasmid, whereas increasing plasmid cost results in intra-specific competition eliminating plasmids by purifying selection, which again reduces the potential for network effects on host dynamics (Figs M and N in S1 Appendix).

### Applying the model to an empirical system with heterogeneous parameters

The plasmid compatibility network effectively blocks potential interactions between hosts and the plasmids they could acquire. Therefore, if there are no ecological differences among plasmids or among hosts, the end plasmid prevalence (but not the subpopulation composition or community dynamics) can be derived directly from the properties of the nodes in the networks (S1 Appendix). Hence, we relaxed the assumption that hosts and plasmids differ only in their position within the interaction networks. To do so, we parameterized the model using an experimental system, thereby introducing heterogeneity in biological parameters and increasing the biological realism of the dynamics. Importantly, our aim was not to empirically validate the model, as such validation would require independent experimental manipulation of infection and compatibility network structures, which is currently not feasible.

**Fig 6:**
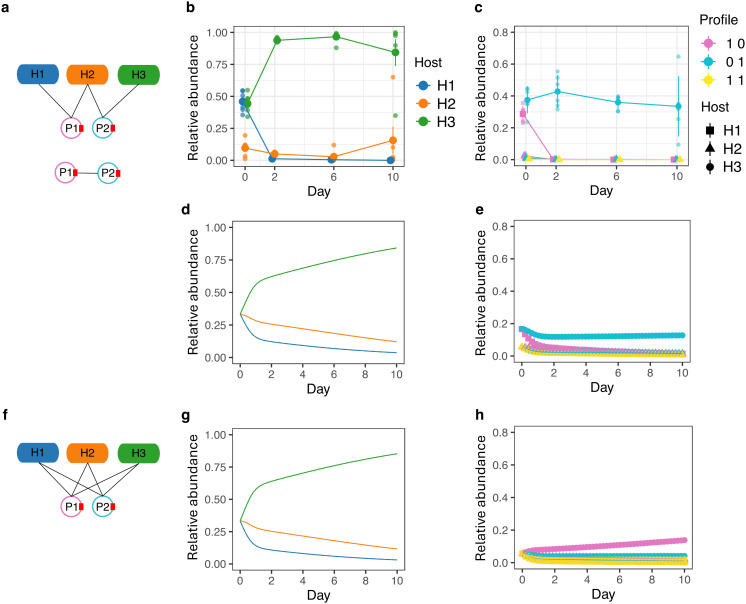
Empirical network structures and simulated population dynamics. **(a)** Empirical infection network **I** (top) and plasmid compatibility network **P** (bottom) from the empirical system. **(b)** Host population dynamics in the empirical system. **(c)** Infected subpopulation (plasmid) dynamics in the empirical system. **(d)** Simulated host population dynamics using the empirical networks. **(e)** Simulated infected subpopulation dynamics using the empirical networks. The empirical and modeling systems show similar patterns for host (panel b vs panel d) and plasmid (panel c vs panel e) dynamics. **(f)** Full infection network **I** used in simulation. **(g)** Simulated host population dynamics under the full infection network. **(h)** Simulated infected subpopulation dynamics under the full infection network. All abundances are relative to community size. Error bars represent SE. The empirical observations had a sample size of 6, while the model simulations had a sample size of 300.

We used data reported in [54], comprising three bacterial host populations and two plasmids distinguishable by their colony phenotypes. The size of this empirical system was constrained by the availability of fluorescent labels used to track the plasmids. The community consisted of *Pseudomonas fluorescens* SBW25 (bacterial host H1), *Pseudomonas putida* KT2440 (H2), and *Escherichia coli* MG1655 (H3). The plasmids were the *Pseudomonas*-specific, mercury-resistant plasmid pQBR57 (P1), and the antibiotic-resistant plasmid pKJK5 (P2), which under the experimental conditions was unable to conjugate into *P. fluorescens* SBW25. The system therefore is characterized by a modular **I** and a full **P** (Fig 6a). Because this empirical system only permits a modular infection structure, we do not have empirical data from the unstructured infection network.

We re-parameterized our model using experimental measurements (Tables A-D in S1 Appendix). Specifically, we incorporated unequal population growth rates (H1 ≤ H2 < H3), infection rates (H1 = H2 < H3), and plasmid costs (P1 < P2), and a heterogeneous interspecific competition network (H1 < H2 < H3). For computational tractability, we used lower values of community-wide carrying capacity ($K_{model}=10^{5}$ vs $K_{empirical}=10^{9}$) and initial abundances ($B_{0model}=3.3\times 10^{3}$ vs $B_{0\mathrm{empirical}}=3.3\times 10^{5}$ for each population). These scaling differences did not affect the qualitative outcomes, and we therefore compared empirical and model results in relative terms.

We then compared model outcomes to the empirical dynamics. Following Fig 3(iii), we expected that in the experimental system the bridge host H2 would be outcompeted by the peripheral hosts and go extinct, and that the resulting community would be primarily composed of infected subpopulations. In contrast to this prediction, H2 maintained a higher relative abundance than the peripheral host H1 (Fig 6b). The most abundant plasmid in the system was P2, mirroring the high abundance of its primary host (Fig 6c). This discrepancy between theoretical expectations and experimental observations can be explained by the heterogeneous growth rates, the interspecific competition network, and an elevated growth rate of the bridge host. We therefore extended the parameterization to explicitly incorporate these factors. When included (Table A in S1 Appendix), the simulations qualitatively reproduced the experimental results (Fig 6b-e; Fig O in S1 Appendix). These results highlight the influence of trait heterogeneity on the consequences of network structure, underscoring that network effects are contingent on underlying parameter configurations. Exploring how parameter heterogeneity interacts with assumed network structures is therefore an important direction for future work.

Having recapitulated the empirical results with our re-parameterized model, we were then in a position to test our hypothesis using a model that fully relaxes the assumption of uniform trait distributions. We reran the model with an unstructured infection network (full **I**; Fig 6f). While the host population dynamics remained unchanged (Fig 6g), the subpopulation dynamics were qualitatively different. Specifically, the less costly plasmid P1 reached a higher community-wide prevalence instead of P2 (Fig 6h vs Fig 6e). These results reinforce our purely theoretical findings, demonstrating that infection network structure can alter plasmid prevalence even in the presence of substantial biological heterogeneity.

## Discussion

Although ecological networks are known to shape community dynamics, we still know surprisingly little about how network structures operate in real host–plasmid systems [23,26]. Empirical data on infection networks and plasmid–plasmid compatibility networks at a community level remain scarce despite increasing studies of plasmid host ranges and co-infection dynamics. Therefore, the effects of each of those networks, let alone their combination, remain unknown. This work provides a first step toward filling this gap by offering a mechanistic framework for studying how network structure can drive community dynamics. Indeed, our results highlight how the individual and combined effects of host-plasmid infection and plasmid-plasmid compatibility networks determine microbial coexistence and plasmid prevalence, potentially shaping long-term evolutionary trajectories by modulating the effects of selective pressures in the community. Because modeling generates testable hypotheses and highlights the processes most worth measuring, these results can help guide empirical studies toward the interaction patterns most likely to influence plasmid spread and evolutionary potential. Emerging technologies, such as Hi-C [55], can now map these interaction networks at scale, making such targeted investigations increasingly feasible.

A central insight from our work is that the infection network exerts a stronger influence on community dynamics because it is a primary barrier to plasmid-plasmid interaction, generating marked heterogeneity in plasmid prevalence and co-infection patterns across hosts. This asymmetry arises because infection structure determines whether hosts incur plasmid-associated costs at all: hosts that are weakly connected or disconnected in the infection network can avoid plasmid carriage entirely, whereas compatibility structure can only limit additional costs once at least one plasmid is present. Preventing infection is therefore more effective at minimizing fitness costs than restricting co-infection, causing infection structure to act as a first-order filter on plasmid presence and setting an upper bound on the impact of compatibility structure on community dynamics. However, this influence does not operate in isolation: the infection network better maintains its modular structure when the plasmid compatibility network is also structured, revealing that compatibility constraints feed back onto how infection patterns unfold. Structured compatibility also reshapes host population composition and therefore determines where plasmids can co-reside. Such structure–dynamics feedback can affect opportunities for recombination, evolutionary innovation, and the potential emergence of multidrug resistance through horizontal gene transfer among co-infecting plasmids [3,18,56,57]. These model-derived insights are consistent with empirical studies showing that host–plasmid dynamics are governed jointly by direct host interactions and compatibility constraints [22,46,53,58–60] and that structured infection networks were found to co-occur with restricted plasmid transfer pathways, limiting the spread of costly plasmids [26]. Taken together, these results underscore the importance of studying multiple, coupled networks when seeking to understand the ecological and evolutionary consequences of infectious MGEs.

Our results further indicate a clear asymmetry between host and plasmid dynamics: plasmids are far more likely to persist than their hosts under alternative network structures, provided they can maintain themselves in just one surviving host population. In other words, whereas there can really only be one successful host owing to niche overlap, multiple plasmids can survive in that successful host. This asymmetry emerges because shifts in plasmid compatibility can impose high infection burdens that drive hosts to extinction, whereas changes in infection patterns rarely eliminate plasmids outright as long as any suitable host remains. Consequently, plasmids retain substantial potential for persistence, evolution, and host-range expansion even under reduced host diversity, consistent with empirical observations of broad-host-range plasmids [61].

Plasmid interactions via epistasis (non-additive cost), which is constrained by the compatibility structure, can also affect dynamics. While our model implemented a slight positive epistasis among plasmid costs, a stronger positive epistasis could hinder host coexistence but enhance plasmid co-infection: when the joint cost of carrying multiple plasmids is alleviated, hosts grow faster and compete more strongly within the community, while plasmids coexist more readily within the same host, increasing opportunities for recombination and horizontal transfer. A pure additive effect of plasmid co-infection, on the other hand, will hinder both host coexistence and plasmid persistence, for co-infected host subpopulations will quickly be out-competed.

In contrast to plasmid compatibility networks, infection networks in our model were inherently unstable, frequently leading to host extinction—even though transient, ultimately unsuccessful host populations could still shape the long-term dynamics of plasmid persistence. This contrasts with natural systems, where modular and nested structures are commonly observed [35,38,62], suggesting stabilizing factors for the network structures. Population-level trait heterogeneity and positive epistasis in plasmid fitness costs [8,17], among other possible factors, may mitigate destabilization, allowing structured networks to persist in nature.

### Limitations and future directions

Our study was designed as a first mechanistic step toward understanding how the interplay between host–plasmid infection and plasmid–plasmid compatibility networks shapes community dynamics. To isolate the consequences of network structure, we adopted a simplified framework in which hosts and plasmids differed only in their network position, while biological traits were held uniform. This abstraction allowed us to attribute differences in host coexistence, plasmid prevalence, and non-additive outcomes directly to network architecture. While under uniform traits, plasmid prevalence can be derived analytically from network topology in some cases, subpopulation composition and transient dynamics cannot, because they depend on infection rates, segregation, and demographic stochasticity (S1 Appendix). The simulation framework is therefore necessary for capturing the dynamical and stochastic processes that govern community outcomes, and becomes indispensable when both networks are structured simultaneously and their joint effects on host abundances render even prevalence analytically intractable.

Several assumptions define the scope of our conclusions and point toward natural extensions. *First*, we assumed uniform host and plasmid traits, excluding biological heterogeneity in growth rate, competitive ability, conjugation rate, host range, and carriage cost. Incorporating such heterogeneity could alter our predictions [24,63]. The framework we present is readily extensible to incorporate biologically realistic trait variation and ecological trade-offs. Indeed, parameterizing our model with empirical data did not qualitatively change the key result that network structure alters plasmid dynamics. *Second*, we focused on small, fixed network motifs to maximize interpretability and permit systematic comparison across factorial structure combinations. Natural microbial communities are considerably larger and more complex; functional redundancy and resource specialization in larger systems could dampen the effects of network interplay [64–67]. Scaling the framework to empirically derived networks will be necessary to assess how the principles identified here generalize. *Third*, we treated the infection and compatibility networks as fixed boundary conditions, whereas in reality they emerge and evolve via eco-evolutionary processes [12,23,38]. Host adaptation and plasmid evolution shape infection structure [36,23], while compatibility networks likely evolve through recombination and mutations modulating incompatibility functions [3,48]. Future models could incorporate such evolutionary feedback, shifting from fixed to dynamic, emergent network structures. *Fourth*, we excluded beneficial accessory genes (e.g., antibiotic resistance), which can shift host–MGE interactions from antagonistic to mutualistic [54]. Integrating eco-evolutionary feedbacks and context-dependent plasmid benefits represents an important next step.

Empirical progress will also be essential. Reconstructing host–plasmid infection networks at community scale is increasingly feasible [68,69], and Hi-C technologies [55] are accelerating this effort. Assembling plasmid–plasmid compatibility networks remains more challenging, as it requires identifying molecular mechanisms of co-residence or conducting extensive co-infection experiments. Ultimately, fully understanding the role of plasmid incompatibilities in shaping host coexistence will require combining network inference, controlled experiments, and dynamic modeling across scales—an agenda for which the present study provides an initial framework.

## Conclusion

In conclusion, our study provides a novel perspective on microbial ecology by explicitly demonstrating that the interplay between host-plasmid infection and plasmid-plasmid compatibility networks profoundly shapes community dynamics and evolutionary potential. We find that the interconnectedness of these ecological networks itself stabilizes host-plasmid communities and alters their dynamics, underscoring the need to move beyond studying interaction types in isolation. Beyond microbial ecology, these theoretical and modeling developments offer insights into community dynamics of infectious agents and their hosts.

## Materials and methods

### Host-plasmid model

ODE-based models have been widely used to study host–plasmid dynamics [7–9], but they are less suited to the specific questions we address here. Our focus is on subpopulation-level dynamics, explicit representation of hosts carrying different plasmids, and incorporating demographic stochasticity [70]. It is also more straightforward to embed heterogeneous network structures within an agent-based model. For these reasons, we use a stochastic agent-based model that captures subpopulation dynamics while allowing the network structures to be represented directly. Below are the overview (entities, spatial and temporal scales), design (events, simulation) and detail (interaction networks, host and plasmid traits, infection propensity, rates, and model limitations) [71].

### Entities

The entities of the model were host subpopulations. Each subpopulation of host *i* contained a plasmid profile *p*. Each plasmid profile was a vector of elements 0 and 1, representing the presence (1) or absence (0) of each plasmid in the subpopulation. Therefore, a community with *n*_*b*_ hosts and *n*_*p*_ plasmids had at most $n_{b}\times 2^{n_{p}}$ subpopulations. We defined *H*_*i*,*p*_ as the abundance of a subpopulation, and *H*_*i*_ as the abundance of host *i* ($H_{i}=\Sigma_{p=0}^{2^{n_{p}}-1}H_{i,p}$). For simplicity, we assumed each plasmid had a single copy in each host individual. We present an example for this notation in Table 1. Based on the plasmid profiles, we generated a list of donors (the plasmid-infected subpopulations that can transmit plasmids to others), and for each donor a list of recipients (the subpopulations that can receive plasmids from the donor) during model initialization. The lists of donors and recipients were used to sample subpopulations that undergo HGT and were updated during the simulations when new subpopulations emerged.

**Table 1:** Example of entity notation. In this example there are hosts (H1 and H2) and two plasmids (P1 and P2).

Host (*i*)	Plasmid profile (*p*)	Abundance (*H*_*i*,*p*_)
1	[0,0]	10
1	[1,0]	5
1	[0,1]	0
1	[1,1]	0
2	[0,0]	10
2	[1,0]	5
2	[0,1]	0
2	[1,1]	0

### Spatial and temporal scales

We did not consider a spatial structure. We used hour as the time unit, as most per-capita rates are quantified with this unit in microbial studies. We used a 20000 hours time span for the simulations of the theoretical part to ensure stable coexistence, and a 240 hours (10 days) time span for simulations supporting the lab experiment, which is ample for most host populations/communities to reach carrying capacity in a lab environment [10].

### Events

Five major events contributed to the dynamics of the entities: death (D), growth (G), segregation (S), competition (C), and infection (I) (Fig 1b). In each death and competition event, the chosen entity decreased its abundance by one. In each growth event, the chosen entity increased its abundance by one. In each segregation event (i.e., growth with segregation error), we assumed plasmid segregation fails, so the plasmid-free entity $H_{i,0}$ increased its abundance by one. By doing so, we assumed a cell division might lose all rather than one of its plasmid, to keep simulation tractable. Relaxing this assumption will not affect the results, for we also assumed the segregation event occurs very rarely, making its influence negligible. With each infection event, the chosen donor entity *H*_*i*,*p*_ infected a recipient entity *H*_*k*,*q*_, turning it into a transconjugant entity *H*_*k*,*r*_. As a result, the donor’s abundance remained the same, the recipient’s abundance decreased by one, and the transconjugant’s abundance increased by one.

### Simulations

We applied the Gillespie algorithm, which includes the following steps:

1. Initialize the system with variable inputs (including parameter values and initial subpopulation abundances) (S1 Appendix and Table F in S1 Appendix), and set time t to zero.
2. Calculate the total rate of the system $R=R_{D}+R_{G}+R_{S}+R_{C}+R_{I}$, which is composed of the total rates of death (see “Rates” for per-capita rates):
  $$\begin{array}{c} R_{D}=\sum_{i,p}\mu_{i,p}H_{i,p}, \end{array}$$ (1)
  growth:
  $$\begin{array}{c} R_{G}=\sum_{i,p}\eta_{i,p}H_{i,p}, \end{array}$$ (2)
  segregation:
  $$\begin{array}{c} R_{S}=\sum_{i,p}\omega_{i,p}H_{i,p}, \end{array}$$ (3)
  competition:
  $$\begin{array}{c} R_{C}=\sum_{i,p}\xi_{i,p}H_{i,p}, \end{array}$$ (4)
  and infection:
  $$\begin{array}{c} R_{I}=\sum_{i,p}\phi_{i,p}H_{i,p} \end{array}$$ (5)
3. Sample the length of a time step $\Delta t=\frac{X}{R}$, where *X* was drawn from an exponential distribution with a mean of 1.
4. Randomly sample an event, with weights proportional to the event’s weight out of the total rate $\left(R_{D}/R,R_{G}/R,R_{S}/R,R_{C}/R,R_{I}/R\right)$
5. Sample the entity. If the chosen event is not infection, randomly sample an entity *H*_*i*,*p*_, with weights according to the subpopulations’ event rates. If the chosen event is infection, first sample a donor entity *H*_*i*,*p*_ from the plasmid-infected subpopulations, with weights according to their infection rates. Then, sample a recipient entity *H*_*k*,*q*_ from the recipient subpopulations that are vulnerable to the donor, with weights according to their abundances. Finally, sample a transconjugant entity *H*_*k*,*r*_, with weights according to the propensity tensor Γ (see “Infection propensity”).
6. Execute the chosen event for the chosen entity, and update the simulation time ($t^{\prime }=t+\Delta t$).
7. Move to step 3 until the simulation time meets the final time. Meanwhile, record the subpopulation abundances when t is equal to or passes desired length of time set for recording the system’s set (5 hours in our case).
8. When the simulation reaches the defined time limit *t* = 20000 or *t* at which the system collapses (all host populations have zero abundance), write the data frame into the output file in SQLite format (S1 Appendix). The output file was used for further analyses.

### Interaction networks

**Fig 7:**
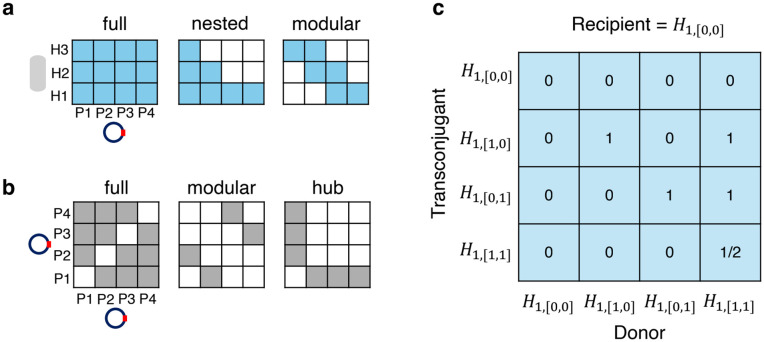
The (a) infection network structures I and (b) plasmid compatibility network structures P represented as matrices. **(c) An example of a propensity tensor.** Colored blocks in (a) and (b) represent blocks with value 0, while white blocks represent blocks with value 1. Quantified structural characteristics of these networks are further displayed in Tables H and I in S1 Appendix. The propensity tensor example focuses on the dimension of the recipient $H_{k=1,q=[0,0]}$. Element $\Gamma_{1,[1,1];1,[1,1];1,[0,0]}=1/2$ because there are two plasmids being transferred from the donor $H_{i=1,p=[1,1]}$ to the recipient $H_{k=1,q=[0,0]}$, creating the transconjugant $H_{k=1,r=[1,1]}$. Each column of the propensity tensor (i.e., with a given combination of donor and recipient) with at least one element >0 was then normalized to 1, ensuring the propensities of each column summed up to 1.

We used an infection network and a plasmid compatibility network. The infection network **I** was a binary incidence matrix (Fig 7a) that determined if the plasmid β (column) can infect the host *i* (row). The plasmid compatibility network *P* was a symmetric binary matrix (Fig 7b) that determined if two plasmids α and β can coexist within the entity’s individuals, with the assumption that plasmids were self-incompatible ($P_{\alpha \beta }=0\;\forall \;\alpha =\beta$). We also assumed that the hosts had equal strength of interspecific competition for a common resource, and that HGT occurred both between and within hosts.

### Host and plasmid traits

For the host, we used per capita growth rate $\eta_{i}$, per capita death rate $\mu_{i}$, and probability of segregation error *e*_*i*_. For the plasmids, we used plasmid cost on host growth $c_{\alpha }$. We applied a community-wide carrying capacity *K*, limiting the sum of population abundances. A complete list of parameters and their values is provided in Table 2 [72].

**Table 2:** Parameter values used in the simulations of the theoretical part. Parameter values were based on/taken from [72].

Host traits	H1	H2	H3	
growth rate $\eta_{i}$	1	1	1	
death rate $\mu_{i}$	0.12	0.12	0.12	
infection rate $\gamma_{i}$	10^−5^	10^−5^	10^−5^	
segregation rate *e*_*i*_	10^−8^	10^−8^	10^−8^	
community-wide carrying capacity *K*	20000	20000	20000	
intraspecific competition coefficient *a*_*ii*_	1	1	1	
interspecific competition coefficient *a*_*ij*_	0.01	0.01	0.01	
Plasmid traits	P1	P2	P3	P4
plasmid cost $c_{\alpha }$	0.3	0.3	0.3	0.3

### Infection propensity

When the infection event is chosen, we considered the propensities with which transconjugants were generated given combinations of donors and recipients. Specifically, we used a three-dimensional infection propensity tensor Γ, with each dimension of size $n_{b}^{n_{p}}$ corresponding to all potential entities (subpopulations) *H*_*i*,*p*_ along the transconjugant, donor, and recipient axes. We assumed that the more plasmids transmitted in an infection, the lower the propensity. Thus, the elements (propensities) of Γ that met the infection conditions (Table 3) were estimated following the power law:


$$\begin{array}{c} \Gamma_{k,r;i,p;k,q}=\frac{1}{2^{\nu -1}}, \end{array}$$ (6)

**Table 3:** Conditions for infection to occur.

Condition	Content
1	Donor is plasmid-infected (p≠0)
2	Donor has plasmid(s) that the recipient does not
3	Transferable plasmid(s) can infect the recipient (${𝐈}_{\beta i}=1$)
4	Plasmids in the transconjugant can coexist (${𝐏}_{\alpha \beta }=1$)

Here, $\Gamma_{k,r;i,p;k,q}$ represented the propensity of the recipient *H*_*k*,*q*_, after receiving plasmid(s) from donor *H*_*i*,*p*_, to become transconjugant *B*_*k*,*r*_. The parameter ν represented the number of plasmid strains being transferred from the donor to the recipient. Other elements of Γ were treated as 0. We provide an example propensity tensor for a system with one host and two plasmids in Fig 7c.

### Rates

The dynamics of the subpopulations (S1 Appendix and Fig P in S1 Appendix) were based on a modified Lotka-Volterra model with infection-recovery elements and direct competition events (individuals killing each other due to competition over a common resource; see S1 Appendix and Figs Q-T in S1 Appendix for results from indirect competition, i.e., density-dependent growth). In this model, each subpopulation had its per capita rate of events based on strain and plasmid profile, and its total rates of events based on its per capita rates × abundance, which summed up to the total rate of events *R*. Below we define the equations of subpopulation per capita rates (variables used in the equations are described in Table E in S1 Appendix). We defined the subpopulation per capita death rate as $\mu_{i,p}=\mu_{i}$ (host-specific) and the per capita growth rate as


$$\begin{array}{c} \eta_{i,p}=\eta_{i}\prod_{\alpha |p_{\alpha }\neq 0}(1-c_{\alpha }), \end{array}$$ (7)

where the realized growth rate was the host-specific per capita growth rate, $\eta_{i}$, times the product of the complements of the costs across plasmids hosted by the subpopulation. Therefore, we assumed that the plasmid costs are not additive but multiplicative for the host, resulting in a slight positive epistasis (as the cost of each plasmid is smaller than 1, the multiplicative cost will be smaller than the pure additive cost). We defined the subpopulation per capita segregation rate as


$$\begin{array}{c} \omega_{i,p}=e_{i}\eta_{i,p}. \end{array}$$ (8)

We defined the competition matrix **A**, in which each cell *a*_*ij*_ is the effect of host *j* on the per-capita growth rate of host *i*. As with other parameters, we used a uniform $a_{ij}=0.01$ to ensure that the community effects we get are not due to some random competitive advantage of one host over another, but rather due to the structure of the networks. We applied a community-wide carrying capacity *K*, and defined the subpopulation per capita competition rate as


$$\begin{array}{c} \xi_{i,p}=\eta_{i,p}\left(\sum_{j}b_{ij}\frac{H_{j}}{K}\right). \end{array}$$ (9)

Here, $b_{ij}=a_{ij}+1$ for i≠j, and *b*_*ij*_ equals to 1 when *i* = *j*.

We defined the subpopulation per capita infection rate $\phi_{i,p}$ as


$$\begin{array}{c} \phi_{i,p}=\gamma_{i,p}\sum H_{i,p}\in 𝐋, \end{array}$$ (10)

where $\gamma_{i,p}$ is the per capita encounter rate between a donor (i.e., plasmid-hosting) and its recipients, and was either host-specific for subpopulations hosting plasmid(s), or zero for the plasmid-free subpopulations. **L** was the set of recipient subpopulations that could receive plasmid(s) from the donor subpopulation. While the mechanisms by which the donors and recipients of plasmids meet could be complex [73], we assumed these mechanisms to be donor-dependent and plasmid-profile-independent.

### Model limitations

Although our model can simulate multihost–multiplasmid systems, it faces computational constraints as system complexity increases. As the number of host (*n*_*b*_) and plasmid populations (*n*_*p*_) increases, so does the time required to generate the infection propensity tensor (of size $n_{b}\times 2^{n_{p}}$ in each of the three dimensions), and to initialize the system (e.g., generate lists of donors and corresponding recipients) unless the interaction networks are extremely sparse. Because the Gillespie algorithm is used, increasing system complexity and community-wide carrying capacity inevitably raises the total event rate *R*. This, in turn, reduces the sampled time step in each iteration, making it more time-consuming for the simulation to reach the final time (S1 Appendix and Fig U in S1 Appendix).

### Experimental design

We used a 3 × 3 factorial design of the combinations of **I** and **P**, which resulted in 9 experiments (Table 4). Due to the stochastic nature of the model, we ran 300 replicates for each experiment, each with a different seed for random sampling. To test how plasmids spread, we initiated populations with a low abundance of monoplasmidic subpopulations (10) compared to plasmid-free subpopulations (2000). We included all potential monoplasmidic subpopulations, so that each plasmid already existed in focal hosts before it was acquired from other hosts.

**Table 4:** Experimental design of the theoretical part.

Expt.(code)	I	P
1 (Full I Full P)	full	full
2 (Full I Mod P)	full	modular
3 (Full I Hub P)	full	hub
4 (Nest I Full P)	nested	full
5 (Nest I Mod P)	nested	modular
6 (Nest I Hub P)	nested	hub
7 (Mod I Full P)	modular	full
8 (Mod I Mod P)	modular	modular
9 (Mod I Hub P)	modular	hub

For the empirical observation, the host-plasmid communities were cultured over five 48-hour transfers (10 days) in shaken liquid medium. We used the empirical data only from the communities that did not experience environmental stress. We used bacterial derivatives and fluorescent proteins to track different host populations and plasmids, and applied a link-balanced initial community composition, where all populations started with equal abundance, comprising of 50% plasmid-free subpopulation and 50% plasmid-carrying subpopulations. We quantified population and subpopulation abundance on days 2, 6, and 10, and summarized population and subpopulation dynamics.

### Code and data availability

The code and data for model simulation, input generation, output analyses, and empirical data analysis is available in the dedicated Github repository associated with this paper at: https://github.com/HFSP-EcoNets/PlasmidNetworkInterplay. Empirical data is also available in the collaborative study by Schaal et al. [54].

## Supporting information

S1 Appendix Supporting information for the host-plasmid model. Combined supplementary file containing: an analytical derivation of plasmid prevalence; subpopulation dynamics; model input and output; results from an alternative model with density-dependent growth rate; complexity-dependent computational efficiency; Figs A–U (supplementary figures referenced in the main text); and Tables A–I (supplementary tables of model parameters, initial conditions, variable definitions, and quantified network properties).
(PDF)
