# Supplementary material for: The interplay between ecological networks drives host-plasmid community dynamics
Source: PLoS Comput Biol. 2026 May 26;22(5):e1014339. doi: 10.1371/journal.pcbi.1014339 (PMC13235927; doi:10.1371/journal.pcbi.1014339)
Supplement: S1 Appendix — Combined supplementary file containing: an analytical derivation of plasmid prevalence; subpopulation dynamics; model input and output; results from an alternative model with density-dependent growth rate; complexity-dependent computational efficiency; Figs A–U (supplementary figures referenced in the main text); and Tables A–I (supplementary tables of model parameters, initial conditions, variable definitions, and quantified network properties). (PDF) [file pcbi.1014339.s001.pdf]

# Supplementary Information: The interplay between ecological networks drives host-plasmid community dynamics

Ying-Jie Wang*, Kaitlin A. Schaal, Johannes Nauta, Armun Liaghat, Manlio De Domenico, James P. J. Hall, Shai Pilosof*

* Corresponding author: lanexran@gmail.com, pilos@bgu.ac.il

**This PDF file includes:**

S1 Appendix
Figs A to U
Tables A to I

# S1 Appendix

Below is the supporting information for the host-plasmid model.

## Analytical derivation of plasmid prevalence

In a system where hosts and plasmids differ only in their position within the interaction networks (i.e., all biological traits are held uniform), the final plasmid prevalence can be derived analytically from the topological properties of the nodes; namely, their degree and the extent of their shared interactions. We provide the derivations below, organized by the results section. We then explain why this analytical tractability does not extend to subpopulation composition, and therefore why simulation remains necessary even in the uniform-trait case.

The plasmid compatibility network **P** constrains which plasmids can co-reside within a host. Combined with the infection network **I**, which constrains which plasmids can infect which hosts, these two networks together define the set of *accessible infection profiles* for each host: the set of plasmid combinations that a host can harbor given both infection and compatibility constraints. When all hosts are ecologically identical and all plasmids share the same transmission parameters and fitness costs, there is no intrinsic advantage to any particular profile. In this case, the long-run expected prevalence of a plasmid $p$ within the surviving host population can be approximated as:

$$\hat{\pi}_p = \frac{|\{\text{profiles accessible to host } h \text{ that contain } p\}|}{|\{\text{all non-empty profiles accessible to host } h\}|} \tag{1}$$

This ratio depends only on network topology and is the same for all hosts under full **I** (since all hosts share the same infection network). We now apply this reasoning to each results section.

#### Section: Structured plasmid compatibility networks promote transient host coexistence

**Full I, full P** Under full **I** and full **P**, each host can acquire any subset of the four plasmids. Excluding the uninfected profile (0000), this yields $2^4 - 1 = 15$ possible infection profiles: the four single-plasmid profiles ($P1$, $P2$, $P3$, $P4$), all $\binom{4}{2} = 6$ pairwise combinations, all $\binom{4}{3} = 4$ three-plasmid combinations, and the fully infected profile $P1$+$P2$+$P3$+$P4$. Each plasmid appears in exactly 8 of these 15 profiles—for example, $P1$ appears in: $\{P1\}$, $\{P1, P2\}$, $\{P1, P3\}$, $\{P1, P4\}$, $\{P1, P2, P3\}$, $\{P1, P2, P4\}$, $\{P1, P3, P4\}$, and $\{P1, P2, P3, P4\}$. The expected prevalence of each plasmid is therefore $8/15 \approx 0.53$, consistent with the simulation results in Fig 2c(i) and Fig 3c(i).

**Full I, modular P** Under modular **P**, plasmids are partitioned into two incompatibility modules: $\{P1, P2\}$ and $\{P3, P4\}$. Each host can therefore harbor at most one plasmid from each module, yielding two mutually exclusive infection routes: a host can acquire either $P1$ and/or $P2$, or $P3$ and/or $P4$. Each route yields three non-empty profiles: $\{P1\}$, $\{P2\}$, $\{P1, P2\}$ for the first; $\{P3\}$, $\{P4\}$, $\{P3, P4\}$ for the second. Within a given route, each plasmid appears in all three profiles ($3/3 = 1$). Because all plasmids share identical infection rates, the two routes are equally likely to be realized, so each plasmid is present in half the host population on average, yielding an expected prevalence of 0.5, consistent with Fig 2c(ii).

**Full I, hub P** Under hub **P**, plasmid $P1$ is compatible with all others, while $P2$, $P3$, and $P4$ are mutually incompatible with one another. The accessible infection profiles are: the four single-plasmid profiles ($\{P1\}$, $\{P2\}$, $\{P3\}$, $\{P4\}$); and the three pairwise profiles involving $P1$ ($\{P1, P2\}$, $\{P1, P3\}$, $\{P1, P4\}$)—yielding $4 + 3 = 7$ non-empty profiles in total. Plasmid $P1$ appears in all 7 profiles, giving an expected prevalence of $7/7 = 1$, consistent with Fig 2c(iii). In contrast, $P2$, $P3$, and $P4$ each appear in exactly 2 profiles—for example, $P2$ appears in $\{P2\}$ and $\{P1, P2\}$, with analogous reasoning applying to $P3$ and $P4$—yielding an expected prevalence of $2/7 \approx 0.29$, consistent with Fig 2c(iii).

#### Section: A modular infection network promotes stable host coexistence and plasmid diversity

**Nested I, Full P** Hosts with more links will experience greater fitness costs. Under this assumption, the host with the fewest links (H3) would be expected to outcompete the others (Fig 3d(ii)). Consequently, the only plasmid compatible with this low-degree host (P1) would reach a prevalence of 1 (Fig 3c(ii)).

**Modular I, full P** Under modular **I** and full **P**, each host has exactly two links in the infection network: H1 is compatible with $\{P3, P4\}$, H2 with $\{P2, P3\}$, and H3 with $\{P1, P2\}$. All hosts are therefore comparable in terms of connectivity. However, H1 and H3 share no plasmids with each other, while both share 50% of their compatible plasmids with H2. This asymmetry places H2 at a disadvantage: being co-infected by both

its compatible plasmids (P2 and P3) imposes a higher cumulative fitness cost than the equivalent co-infection experienced by H1 or H3, because H2's two plasmids are the bridge plasmids shared with both peripheral hosts, making H2 the most frequent co-infection target. This leads to the exclusion of H2 (Fig 3d(iii)). Because each host can only be infected by its two designated plasmids, the accessible profiles per host are limited to three non-empty profiles (two singletons and the co-infected pair), mirroring the per-route logic of the modular-**P** case above. The expected plasmid prevalence can therefore be approximated by each plasmid's degree in the infection network divided by the total number of links, which is equivalent to the profile-counting argument in this symmetric case. This yields expected prevalences of $1/6 \approx 0.17$ for $P1$ and $P4$ (each infecting only one host), and $2/6 \approx 0.33$ for $P2$ and $P3$ (each infecting two hosts), qualitatively consistent with Fig 3c(iii). The quantitative differences resulted from the heterogeneous composition of the hosts' populations.

### Section: Combined structures generate non-additive dynamics

**Modular I, hub P** Under modular **I**, H1 is compatible with $\{P3, P4\}$, H2 with $\{P2, P3\}$, and H3 with $\{P1, P2\}$. When $P1$ is the hub plasmid, it is compatible with all others in **P**, while $P2$, $P3$, and $P4$ are mutually incompatible. The realized infection potential of each host is therefore determined by the joint constraints of **I** and **P**: H3 can acquire both $P1$ and $P2$ (realized degree = 2), while H1 and H2 can each acquire at most one plasmid (realized degree = 1), since their compatible plasmids are mutually incompatible under hub **P**. H3 therefore incurs a higher cumulative fitness cost and is outcompeted by H1 and H2 (Fig 4b,d).

When $P2$ is the hub plasmid instead (Fig 4e–h), the structure shifts: H3 retains access to $P2$ via **I**, but now H2 also gains full access to both its compatible plasmids $P2$ and $P3$, since $P2$ as hub is compatible with $P3$. H2's realized degree therefore equals its full potential degree (= 2). Although H3 also has realized degree 2, it shares only one plasmid with another host, whereas H2 shares plasmids with both H1 and H3, making H2 the host with the most shared interactions and therefore the highest fitness costs. H2 is consequently outcompeted and goes extinct (Fig 4h). This explains why H2 coexists under $P1$-hub (where its realized degree is reduced to 1) but goes extinct under $P2$-hub (where its realized degree reaches 2).

Despite this structural shift, plasmid prevalence patterns remain qualitatively unchanged across the two hub choices (Fig 4c vs 4g): $P3$ remains the most prevalent plasmid in both scenarios. The reason is that in both cases, $P2$ is carried primarily by hosts that incur an additional fitness cost from also carrying $P1$ (under $P1$-hub, by H3; under $P2$-hub, by H3 as well, with H2 going extinct), depressing its realized prevalence relative to $P3$.

However, a precise analytical derivation of plasmid prevalence is not straightforward in this combined-network case. Unlike the single-network cases above, the joint constraints of modular **I** and hub **P** produce asymmetries in host competitive ability that translate into unequal host abundances. Plasmid prevalence depends not only on which hosts a plasmid can infect, but on the relative abundances of those hosts—which are themselves dynamical outcomes of competitive exclusion, infection rates, and demographic stochasticity. For example, $P3$ is more prevalent than $P2$ even when both have the same degree in **I**, because the host primarily acquiring $P2$ (H3) also carries $P1$, incurring higher fitness costs and achieving lower relative abundance. This prevalence asymmetry cannot be read off from network topology alone and requires simulation to quantify.

### Why simulation is nonetheless necessary

The analytical arguments above correctly predict plasmid prevalence from network topology in cases where a single network dominates community dynamics. However, simulation is necessary for two independent reasons. First, even in the uniform-trait case, subpopulation composition cannot be derived analytically from network structure. Subpopulations are formed through a sequential, rate-dependent infection process: a host acquires plasmids one at a time at a rate governed by the infection rate $\gamma$, and loses them through segregation at rate $e_i$. This process has a strong directional bias: once a host acquires one plasmid, it becomes a target for further infections, and profiles with more plasmids are both more likely to be reached over time and more stable once formed, because they act as more potent donors. The profile 1111, for example, requires four sequential infection events, but once established it is the most stable profile and the most potent donor. As a result, highly infected subpopulations are systematically overrepresented relative to what equal-profile-probability would predict. This can be seen directly in the simulation output: under full **I** and full **P**, the 1111 profile dominates the surviving host population composition (Fig 2d(i)), even though it represents only 1 of 15 possible profiles and would thus be expected at proportion $1/15 \approx 0.067$ under equal weighting. The observed dominance of the fully infected subpopulation is a dynamical consequence of the infection process, not a topological one. Subpopulation composition matters because it determines the fitness burden experienced by each host, which in turn drives the coexistence dynamics and extinction patterns that are the primary findings of the paper.

Second, when both networks are structured simultaneously, even plasmid prevalence itself is not trivial to derive. As shown above for modular **I** combined with hub **P**, the joint constraints produce asymmetries in host competitive ability and unequal host abundances, so plasmid prevalence depends on the realized abundance of

the hosts a plasmid can infect rather than on topology alone. The non-additive interaction between **I** and **P** is precisely the regime where static topological arguments fail and dynamic simulation becomes indispensable.

Finally, the *transient* coexistence dynamics (i.e., how long hosts coexist before competitive exclusion) cannot be captured by any steady-state topological argument regardless of trait homogeneity. Coexistence duration is intrinsically a dynamical quantity shaped by the interplay between infection rates, competitive exclusion, and demographic stochasticity, and constitutes a central result of the paper.

## Subpopulation dynamics

The simplified dynamics of plasmid-infected and plasmid-free subpopulations are illustrated in Fig P. Each subpopulation experiences events that lead to an increase or decrease in abundance at subpopulation-specific per capita rates. Note that for plasmid infection, the infection rate only determines the frequency of infection events, while the rate-independent propensity tensor $\Gamma$ determines the propensity at which an individual of a given type of plasmid-carrying subpopulation is produced. The equations and related parameters of the per capita rates and the propensity tensor are described in Materials and Methods.

## Input and output

The raw simulation input is in JSON format and includes the variables described in Table F. The raw simulation output is in SQLite format and includes three tables. Table "bsubabundance" is the table of host subpopulation dynamics. It includes time (`t`), subpopulation id (`subpop_id`), host id, plasmid profile (`p_profile`), and subpopulation abundances (`abundance`). Table "events" includes time (`t`), the accumulated number of growth events (`growth`), death events (`death`), segregation events (`segregation`), competition events (`competition`), and infection events (`infection`). Table "meta" includes the general information about the simulation: `seed`, `key` (the id of a simulation and output assigned by the experiment designer), `job` (the id of the job generated by the HPC), `start time`, `end time`, and `elapsed seconds`.

## Results from an alternative model with density-dependent growth

To explore how density-dependent growth (a typical competitive Lotka-Volterra model) affect the results, we re-defined the subpopulation per capita growth rate as

$$\eta_{i,p} = \eta_i(1 - \frac{H_i}{K}) \prod_{\alpha|p_\alpha \neq 0} (1 - c_\alpha), \tag{2}$$

and removed the competition event. The results were qualitatively identical and quantitatively strengthened: a structured plasmid compatibility network **P** promotes transient host coexistence (Fig Q), while a modular infection network **I** promotes stable host coexistence and plasmid diversity (Figs R and S). Also, network structure can counteract the effects of one another. For example, the hub plasmid P1 at hub **P** failed to be prevalent at modular **I** (Figs S and T). The strengthened patterns were due to the density-dependent growth (indirect competition), which limits population growth more efficiently (without time delay) than direct competition events in a stochastic model. Such indirect rather than direct competition delayed competitive exclusion and thereby enhanced host coexistence.

## Complexity-dependent computational efficiency

To demonstrate how the system complexity limits the computational efficiency of our model, we ran a model performance test across n-host-n-plasmid systems where n ranged from one to six. Each n-host-n-plasmid system was initialized with $n \times (n+1)$ subpopulations, where each host population had a plasmid-free subpopulation with 1000 individuals, and all potential single-plasmid-carrying subpopulations with 100 individuals each. We fixed all the host and plasmid traits across populations, applied the reference (full) structure for all interaction networks, and increased the community-wide carrying capacity $K$ linearly with the initial number of subpopulations (Table G). The elapsed time for the infection tensor $\mathbf{\Gamma}$ generation, initialization and simulation substantially increased with system complexity (Fig U).

## Figs A to U

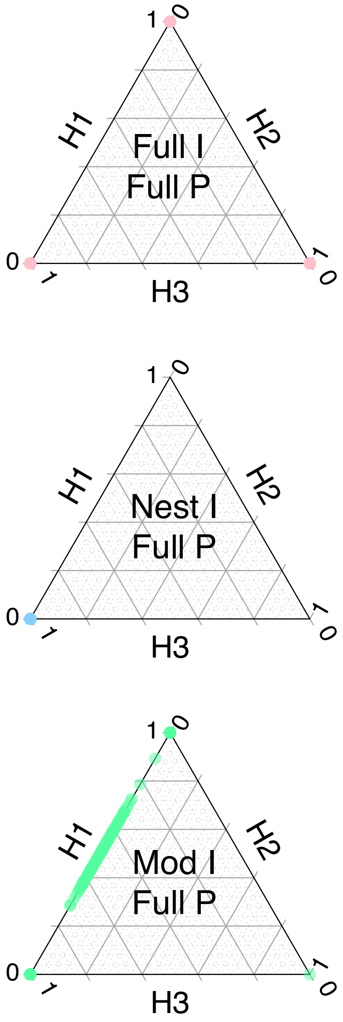


**Fig A.** Relative abundance of populations at the end of the simulations (t = 20000 hours). Dots on the vertices represent no coexistence (i.e. only one population survived), while dots on the edges represent the coexistence of two populations.

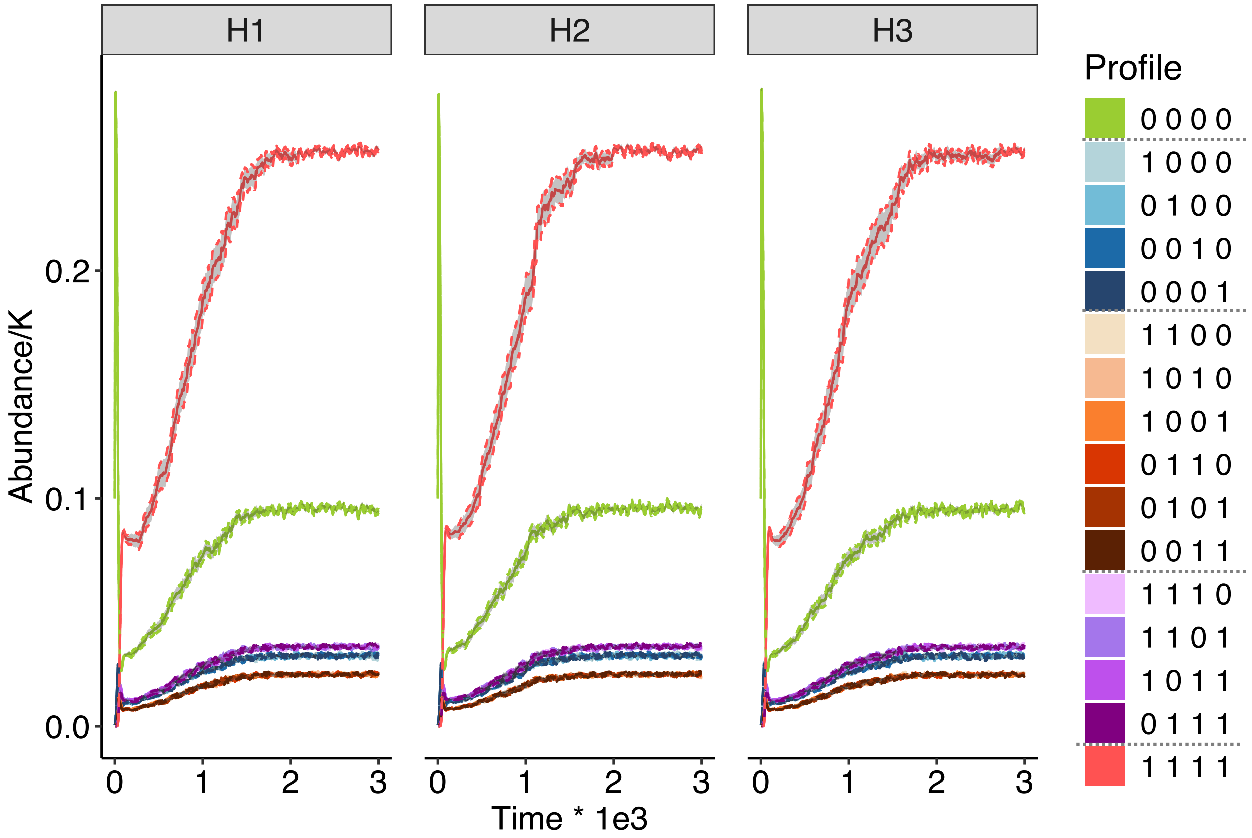


**Fig B.** Dynamics of subpopulation abundance (mean ± 1 SE) at full I x full P. Note that the number of replicates of a subpopulation at a given time point may vary across time, for it depends on how many replicates still have that subpopulation at that time point. The K at y-axis represents carrying capacity. Time was only plotted to t = 3000 where equilibrium of subpopulations had been reached. Note the plasmid-free subpopulation (green line) first increased then decreased.

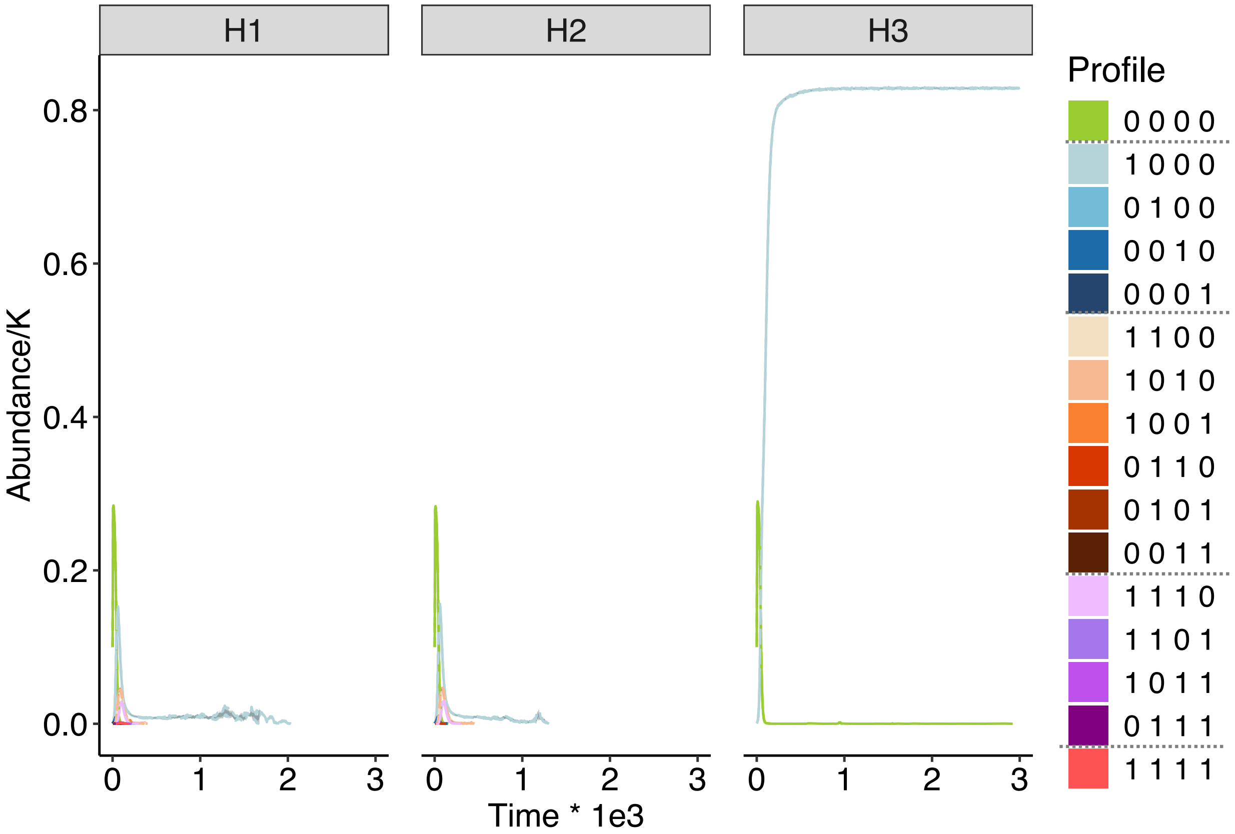


**Fig C.** Dynamics of subpopulation abundance (mean ± 1 SE) at nested I x full P. Note that the number of replicates of a subpopulation at a given time point may vary across time, for it depends on how many replicates still have that subpopulation at that time point. The K at y-axis represents carrying capacity. Time was only plotted to t = 3000 where equilibrium of subpopulations had been reached.

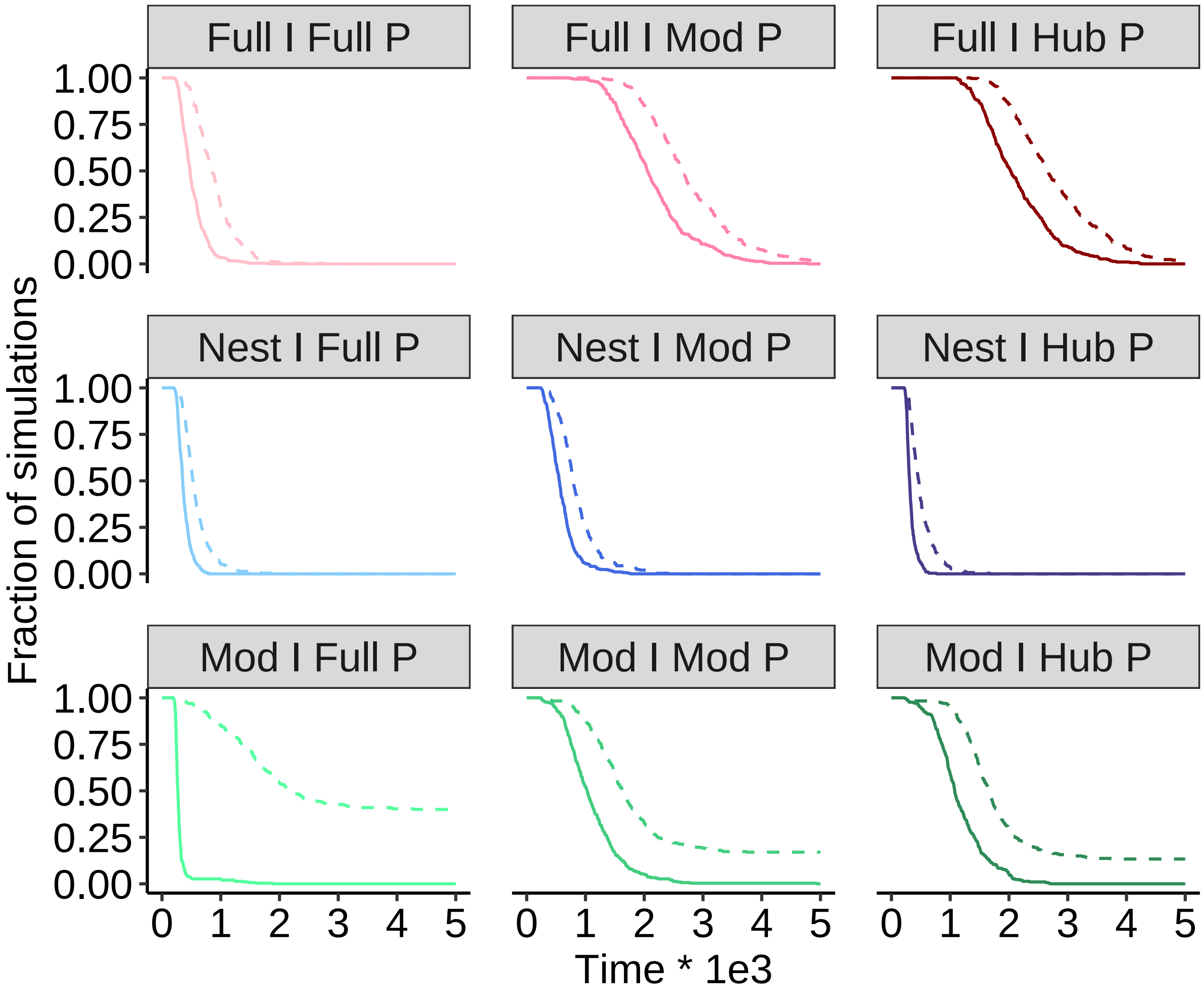


**Fig D.** Dynamics of microbe coexistence of three populations (solid line) and two populations (dashed line) at the end of the simulations (t = 20000 hours). The dynamics of microbe coexistence probability (fraction of simulations) is calculated as the proportion of replicates with complete(3-population)/partial(2-population) coexistence out of total number of replicates across time. Time was only plotted to t = 5000 where all probabilities had dropped to zero.

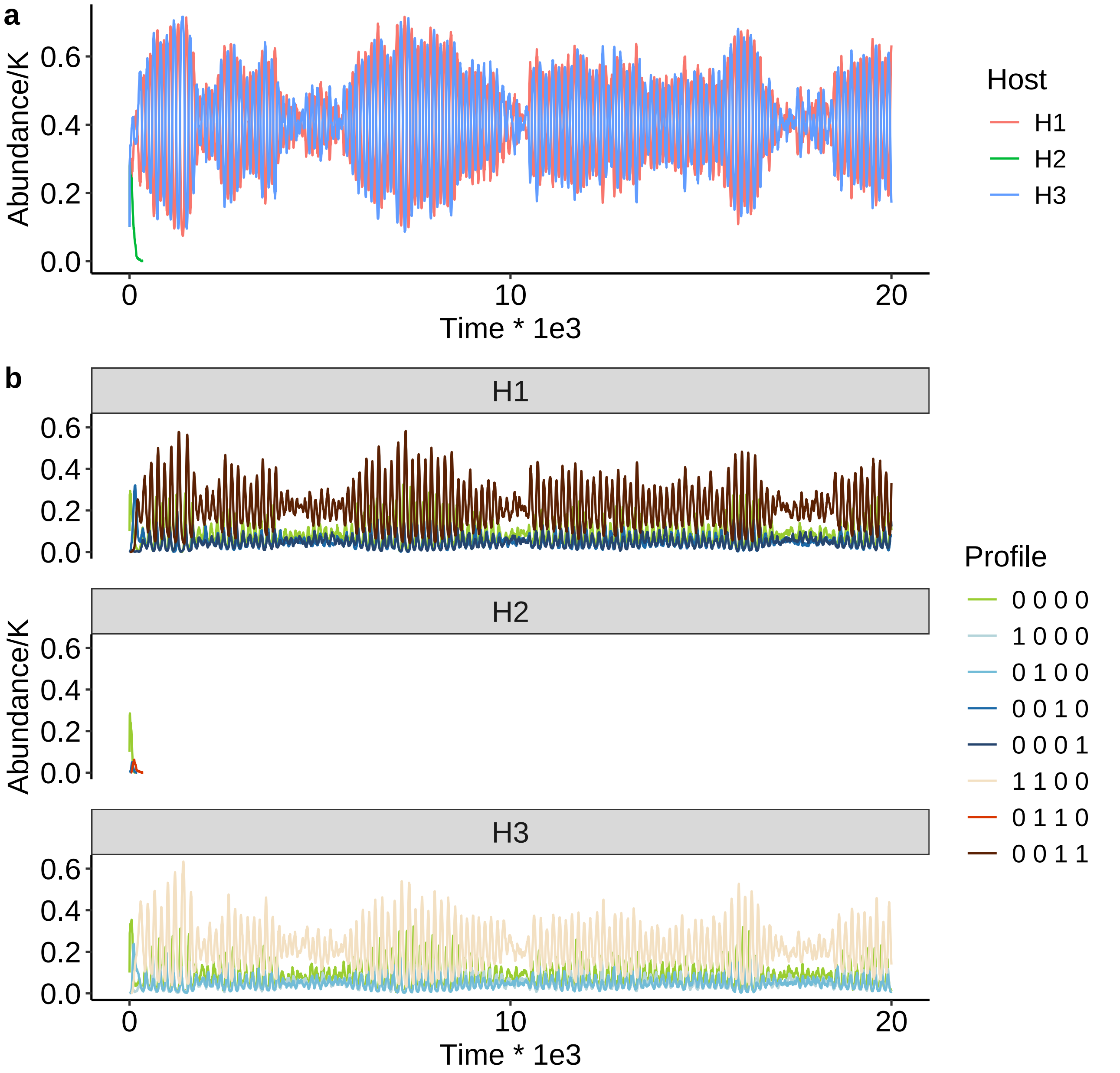


**Fig E.** Dynamics of (a) population abundance and (b) subpopulation abundance at modular I x full P from replicate 21. Replicate 21 demonstrated the stable coexistence scenario where the plasmid-free subpopulations of the peripheral hosts persisted. The K at y-axis represents carrying capacity. Interestingly, populations and subpopulations fluctuated with alternative amplitudes around mean abundance. This is because there was a dynamical balance between primary and secondary plasmid infections between the coexisting hosts.

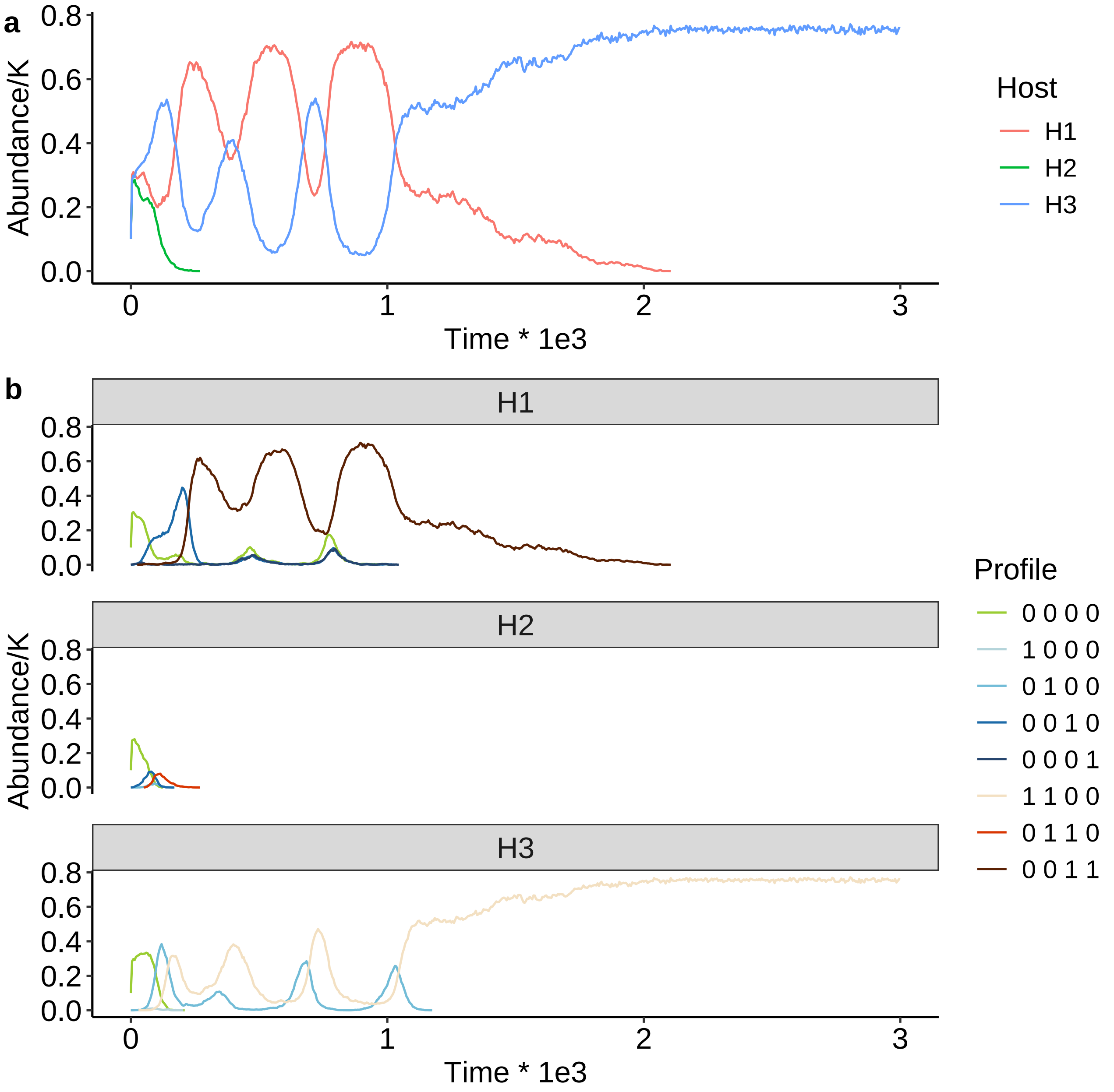


**Fig F.** Dynamics of (a) population abundance and (b) subpopulation abundance at modular I x full P from replicate 23. Replicate 23 demonstrated the unstable coexistence scenario where the plasmid-free subpopulations of the peripheral hosts went extinct. When the plasmid-free subpopulations went extinct, monoplasmidic subpopulations lost input from infection, and the remaining monoplasmidic individuals quickly got co-infected, destabilizing the oscillatory dynamics of the coexisting populations. The K at y-axis represents carrying capacity. Time was only plotted to t = 3000 where equilibrium of subpopulations had been reached.

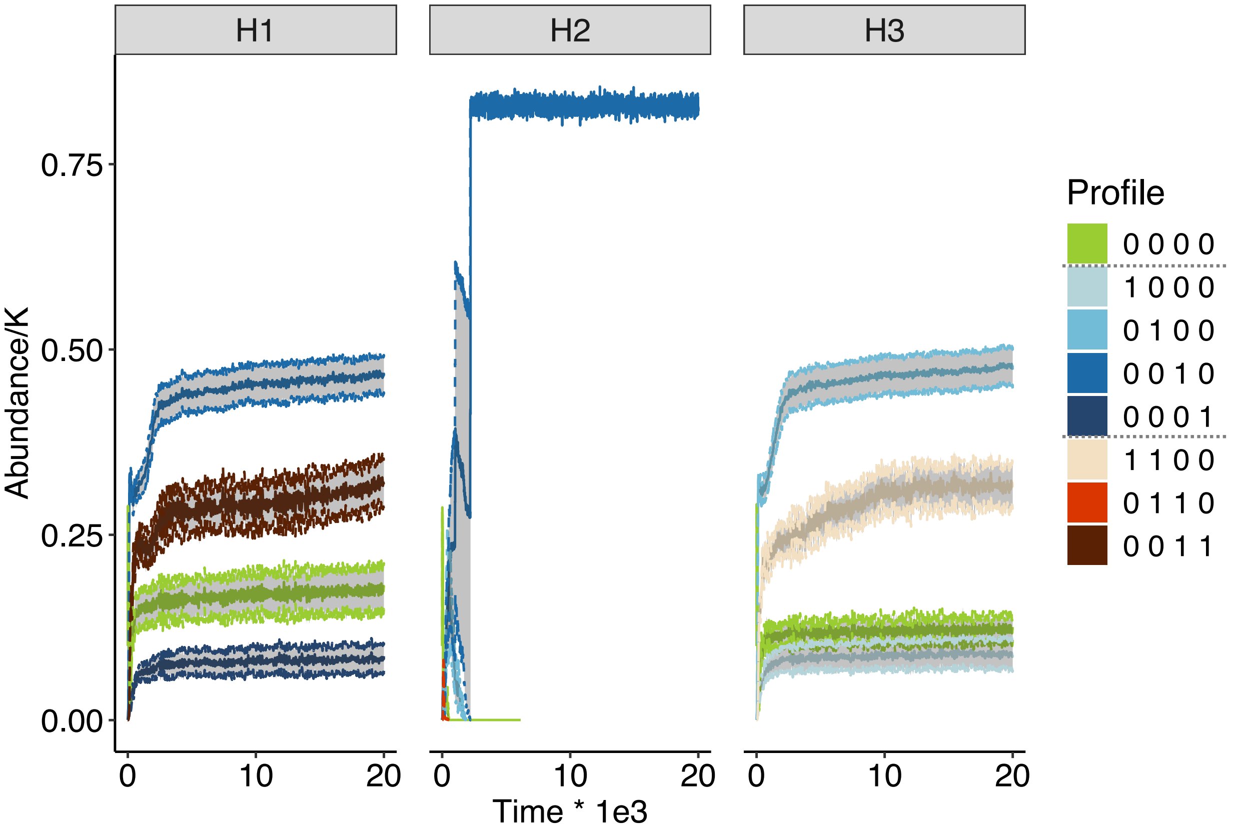


**Fig G.** Dynamics of subpopulation abundance (mean ± 1 SE) at modular I x full P. Note that the number of replicates of a subpopulation at a given time point may vary across time, as it depends on how many replicates still have that subpopulation at that time point. The K at y-axis represents carrying capacity. The high abundance of P3-infected H2 is from a rare case (one from 300 replicates).

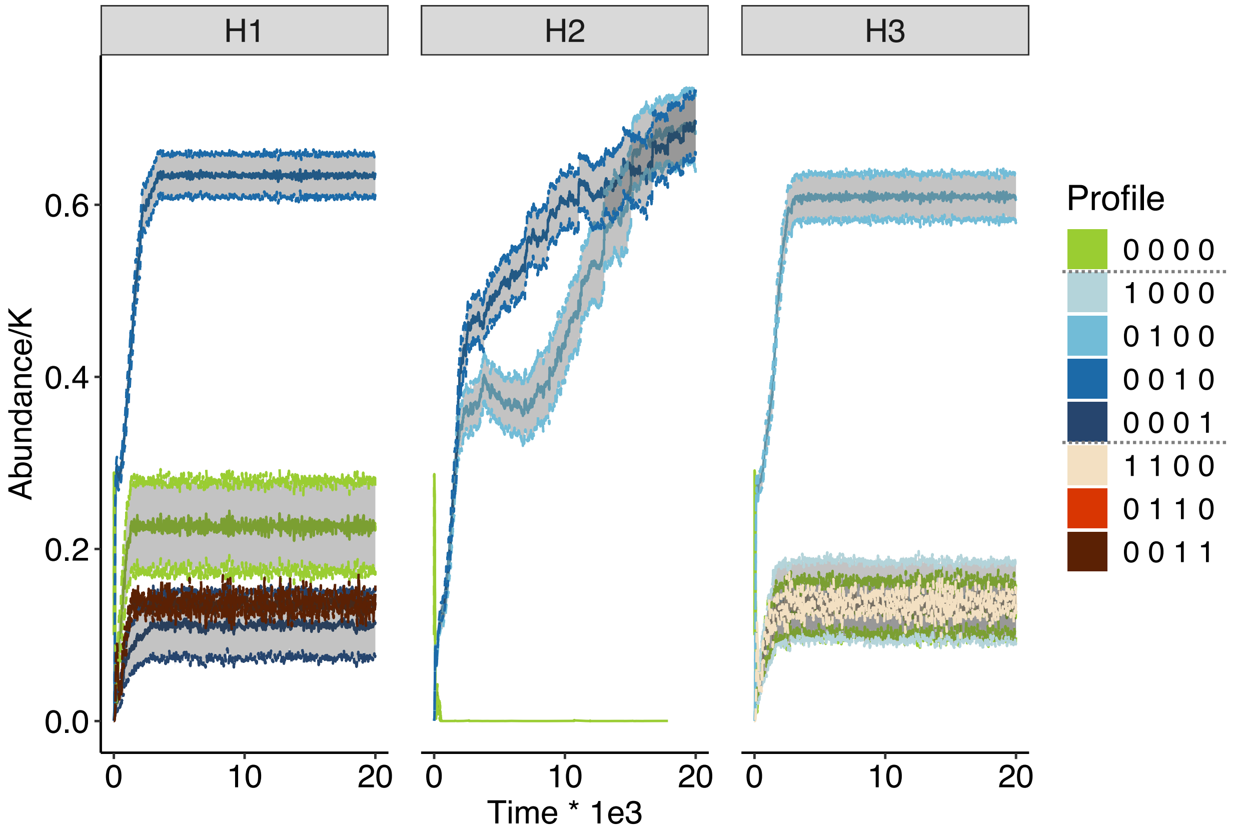


**Fig H.** Dynamics of subpopulation abundance (mean ± 1 SE) at modular I x modular P. Note that the number of replicates of a subpopulation at a given time point may vary across time, for it depends on how many replicates still have that subpopulation at that time point. The K at y-axis represents carrying capacity. The subpopulation abundances of H2 are from 65 replicates.

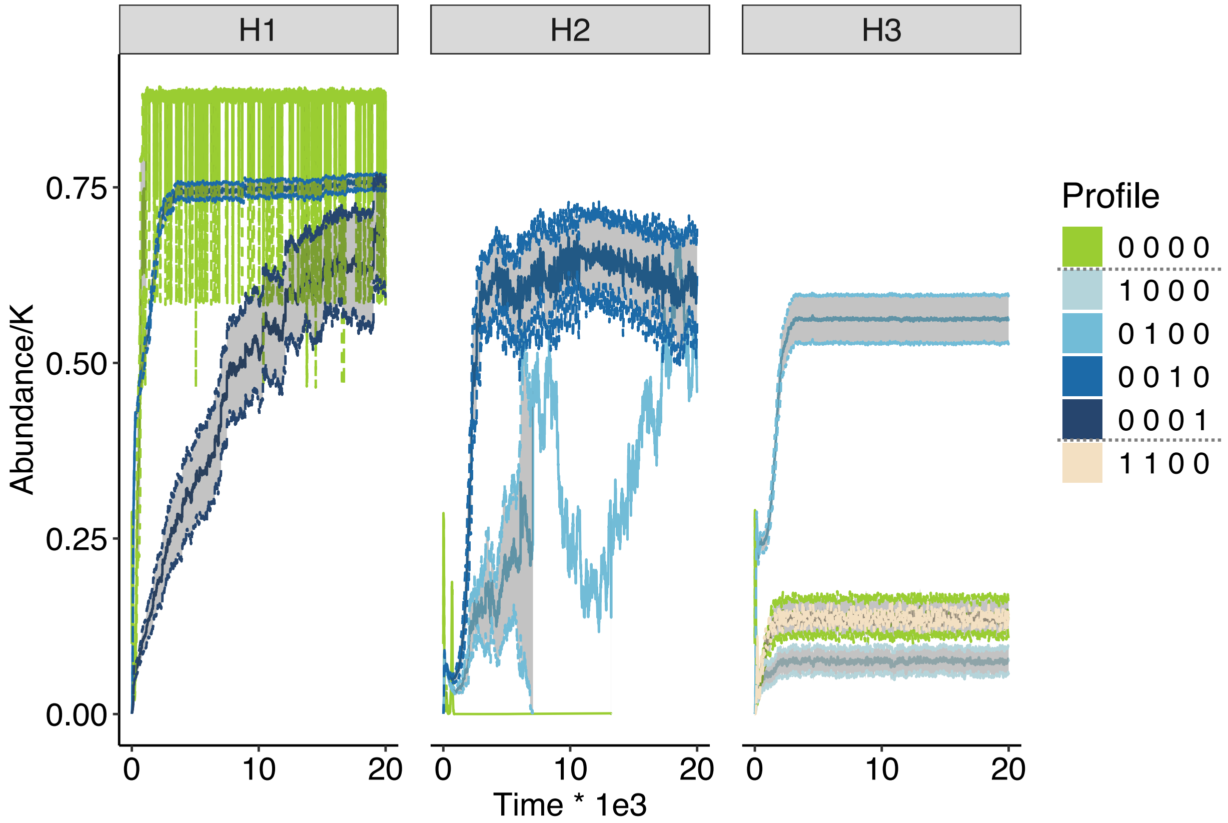


**Fig I.** Dynamics of subpopulation abundance (mean ± 1 SE) at modular I x hub P. Note that the number of replicates of a subpopulation at a given time point may vary across time, for it depends on how many replicates still have that subpopulation at that time point. The K at y-axis represents carrying capacity. The subpopulation abundances of H2 are from 9 replicates.

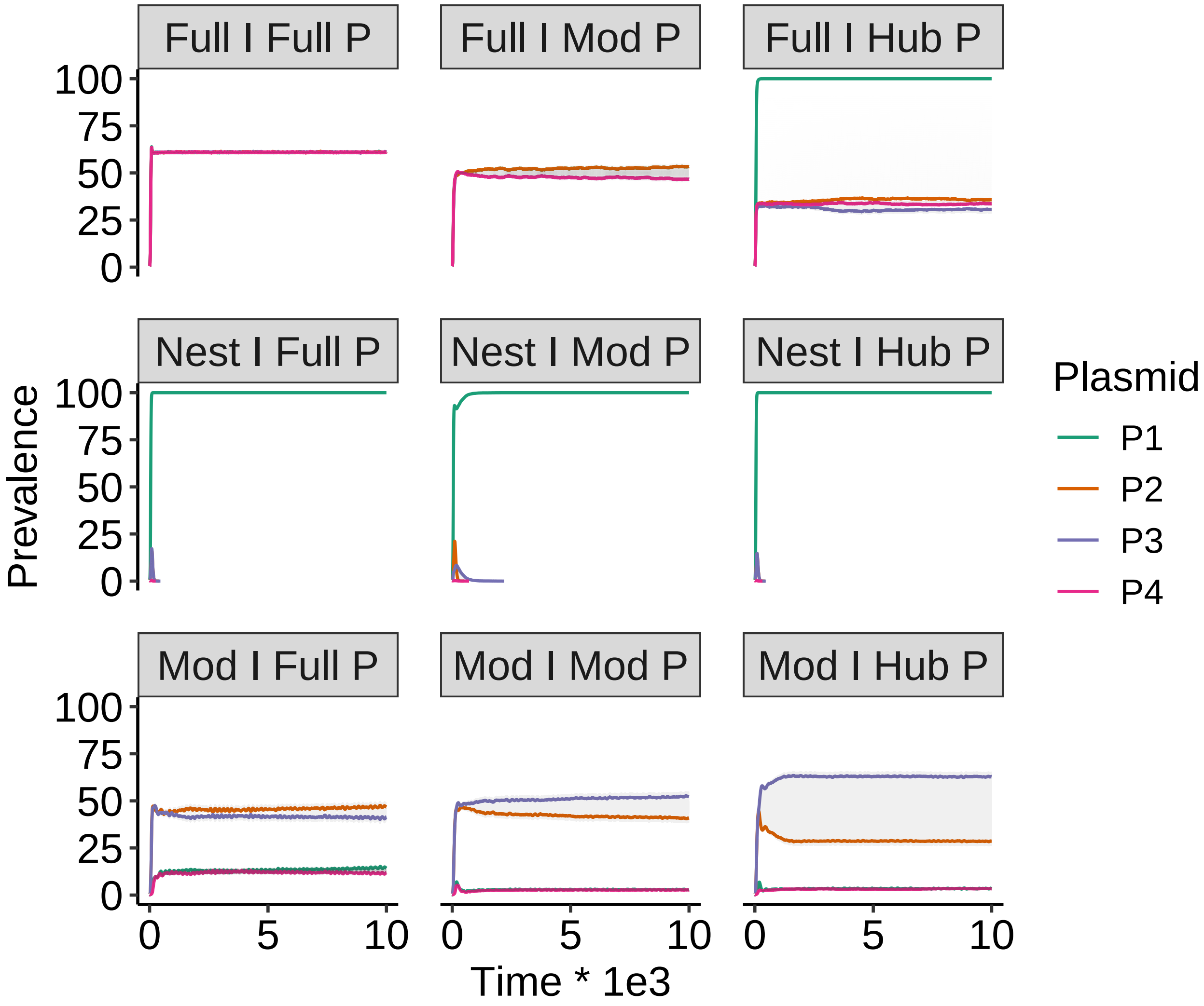


**Fig J.** Dynamics of plasmid prevalence (mean ± 1 SE). Time was only plotted to t = 10000 where equilibrium of plasmid prevalence had been reached.

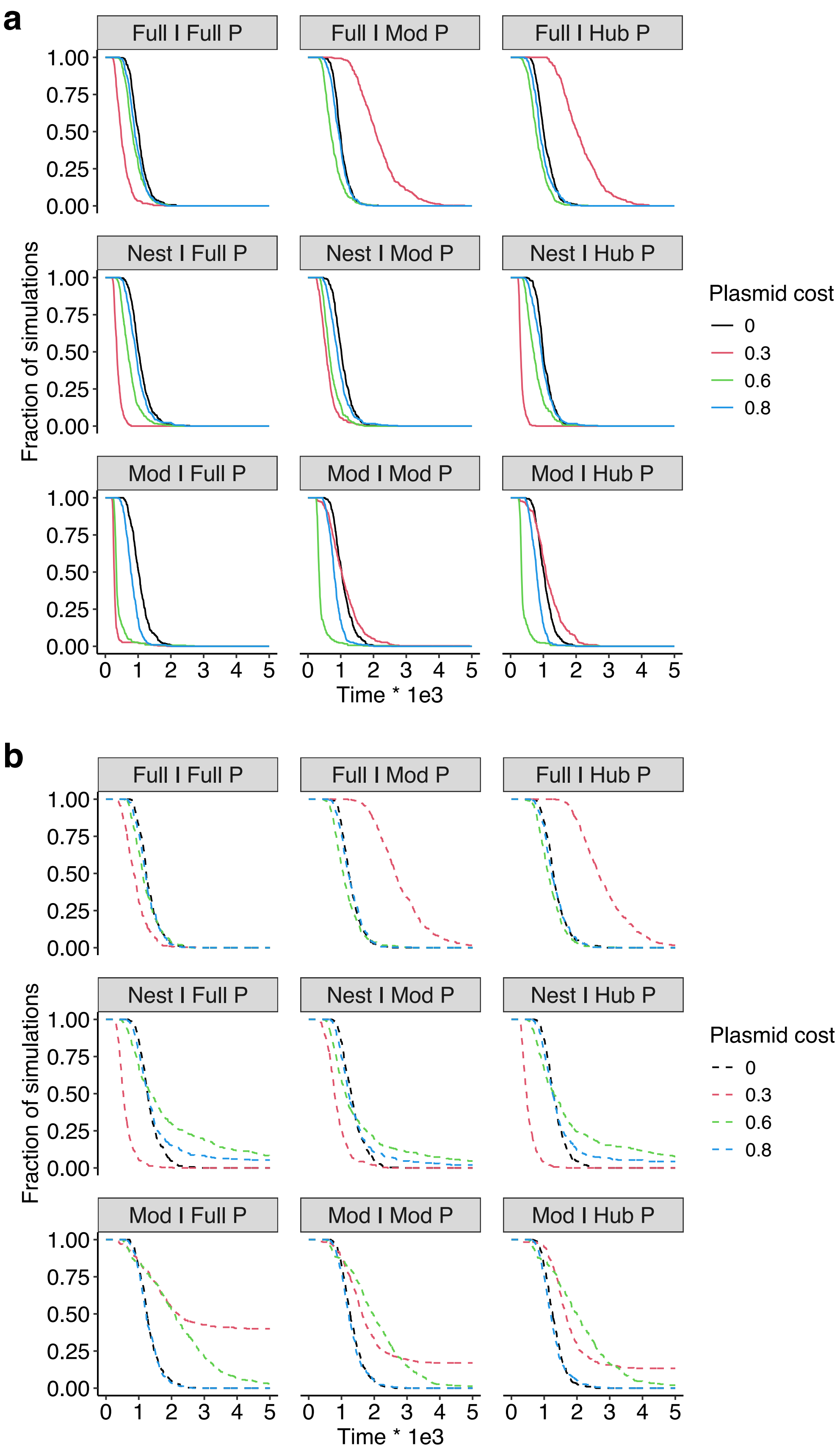


**Fig K.** Dynamics of microbe coexistence of (a) three populations (solid line) and (b) two populations (dashed line) across plasmid costs. The dynamics of coexistence probability (fraction of simulations) is calculated as the proportion of replicates with complete(3-population)/partial(2-population) coexistence out of total number of replicates across time. Time was only plotted to t = 5000 where most probabilities had dropped to zero.

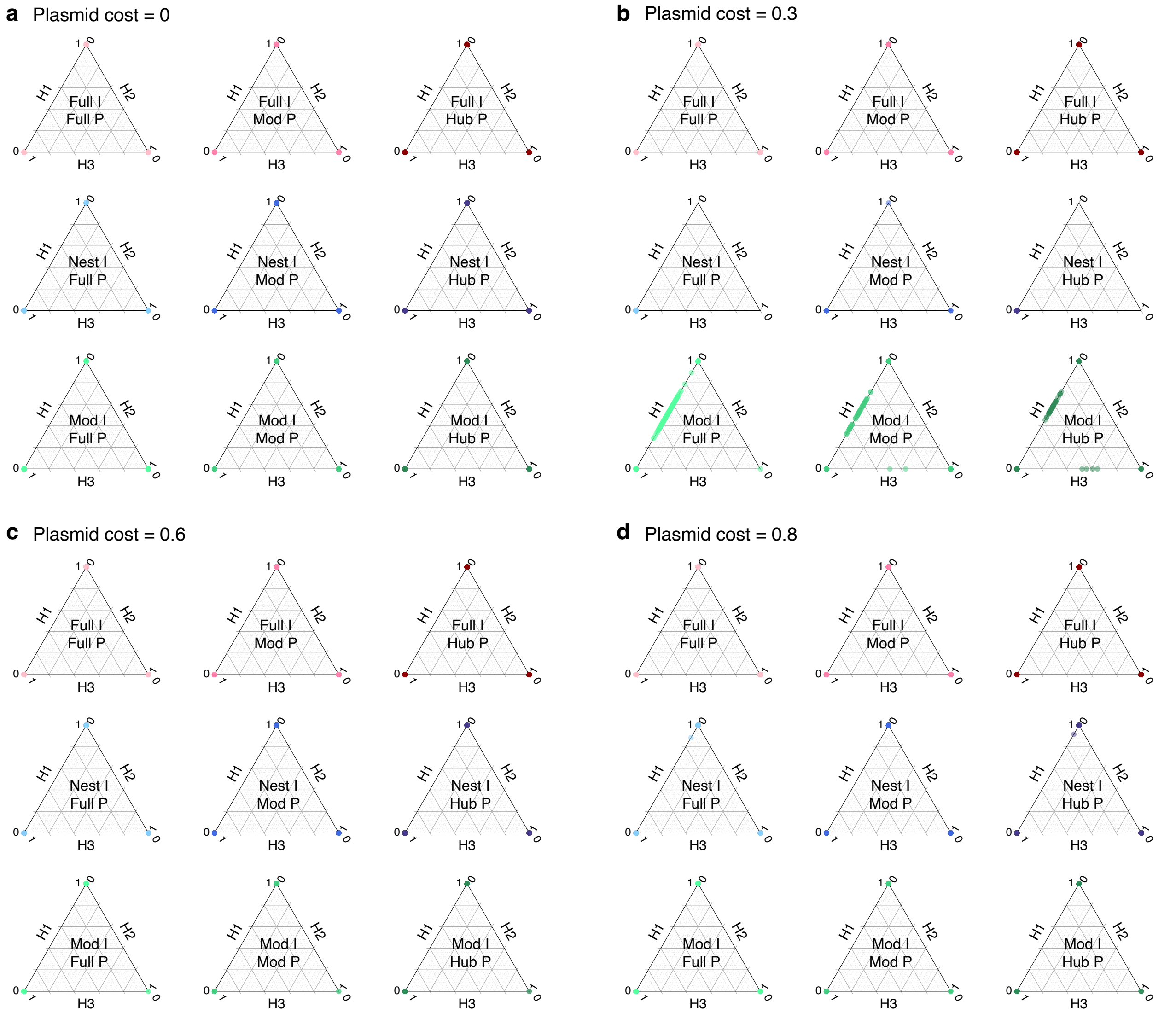


**Fig L.** Relative abundance of populations at the end of the simulations (t = 20000 hours) when plasmid cost = (a) 0, (b) 0.3, (c) 0.6, and (d) 0.8. Dots on the vertices represent no coexistence (i.e. only one population survived), while dots on the edges represent the coexistence of two populations.

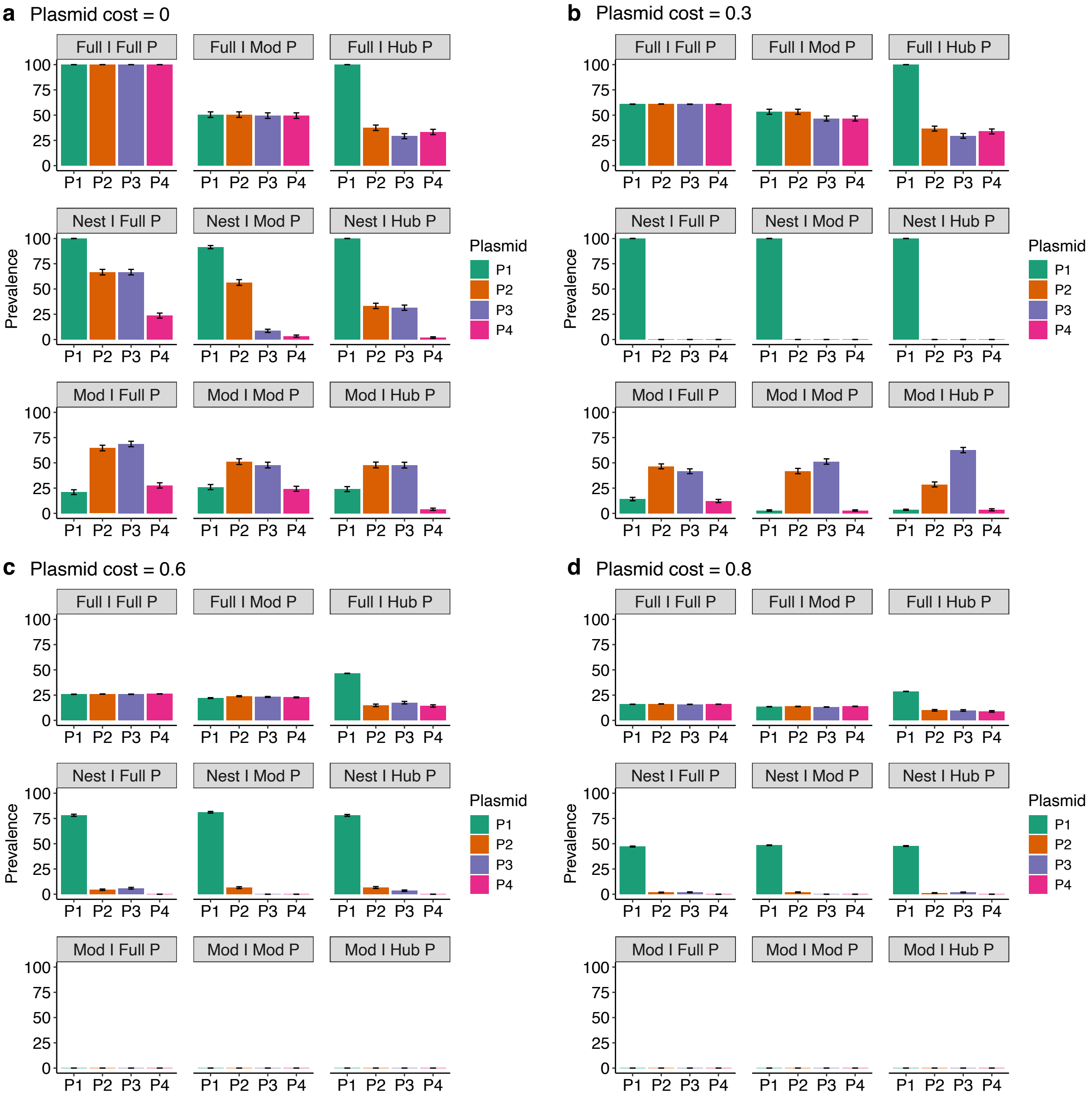


**Fig M.** Mean and SE of final plasmid prevalence across all replicates when plasmid cost = (a) 0, (b) 0.3, (c) 0.6, and (d) 0.8.

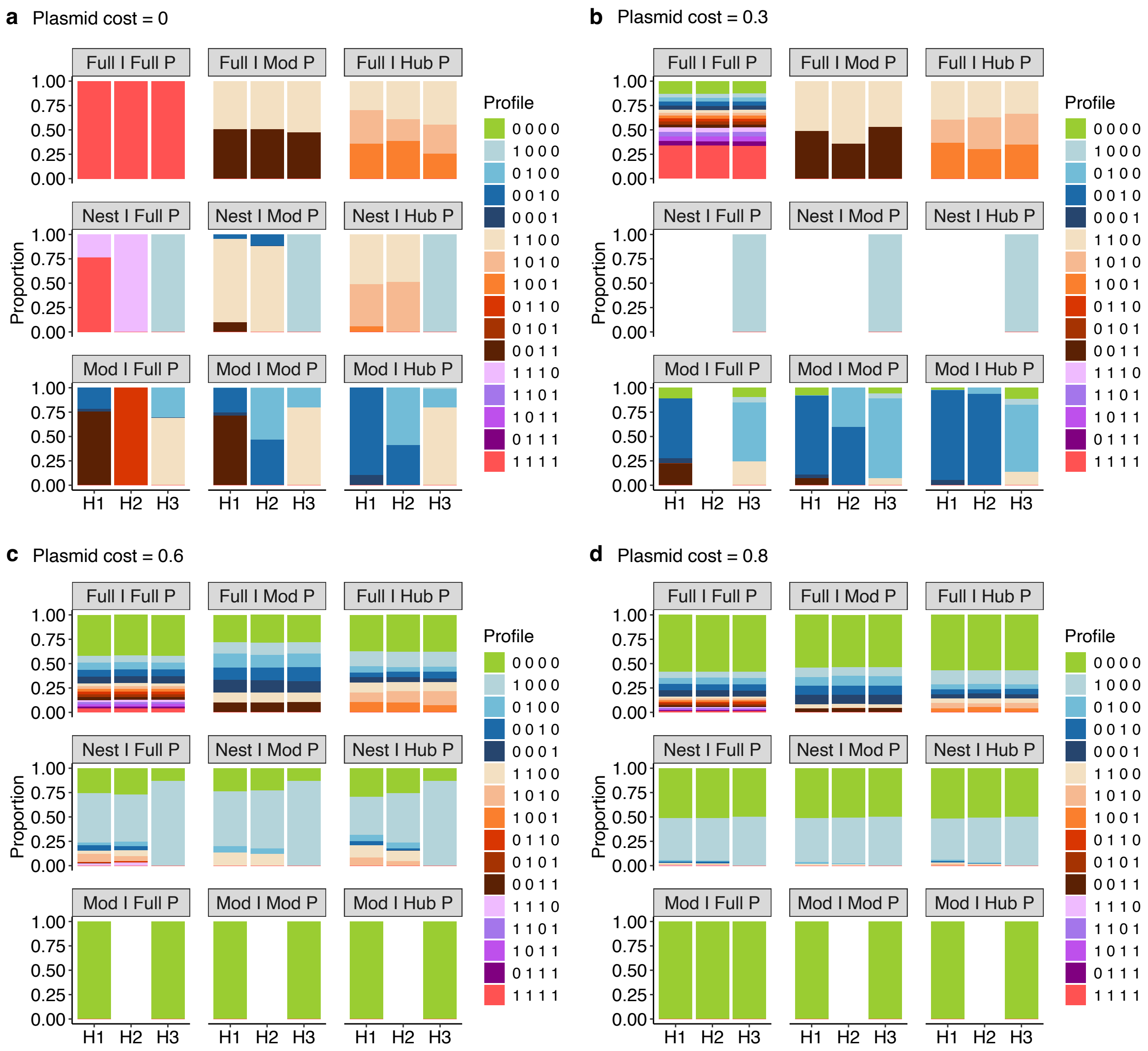


**Fig N.** Final host population composition when plasmid cost = (a) 0, (b) 0.3, (c) 0.6, and (d) 0.8., averaged across replicates in which that host population survived (only populations surviving in > 5 replicates were considered). Profiles represent the host subpopulations (e.g., the profile 1000 represents a subpopulation hosting only P1).

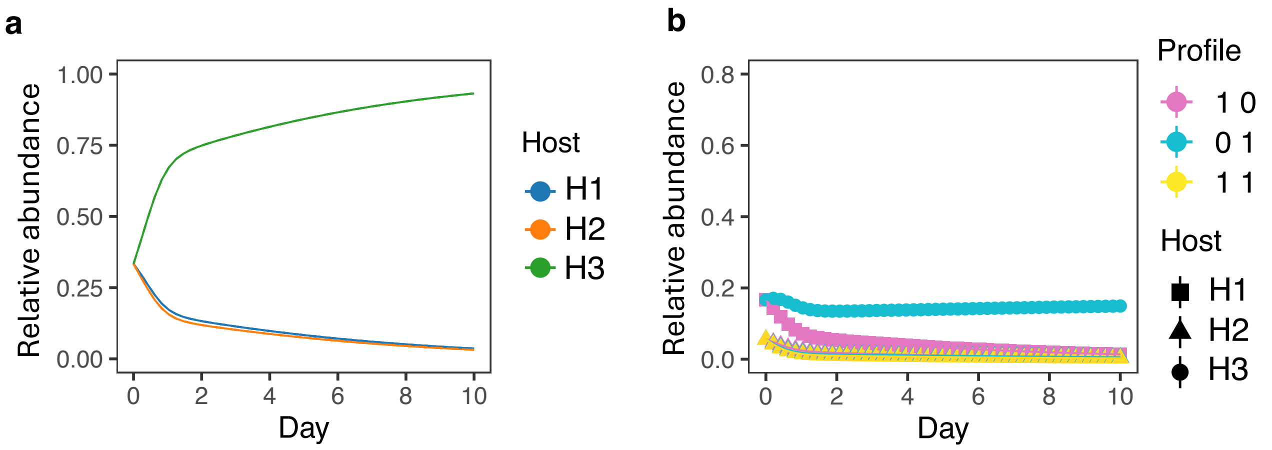


**Fig O.** (a) The host population dynamics and (b) host subpopulation dynamics from the simulations assuming no increase on the growth rate of the bridge host H2 under a modular **I** based on an empirical system (Fig 6a, top row).

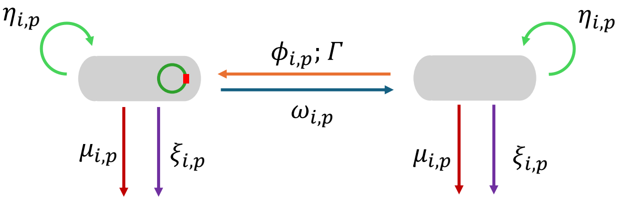


**Fig P.** Illustration of the dynamics of plasmid-carrying subpopulation (left box with a circle in it) and plasmid-free subpopulation (right box). Colored arrows indicate abundance inflows/outflows from the five events: growth (green), death (red), competition (purple), infection (orange), and segregation (blue). Alongside each arrow lists the per capita rate of the event, with subscripts denoting host $i$ and plasmid profile $p$ of the subpopulation.

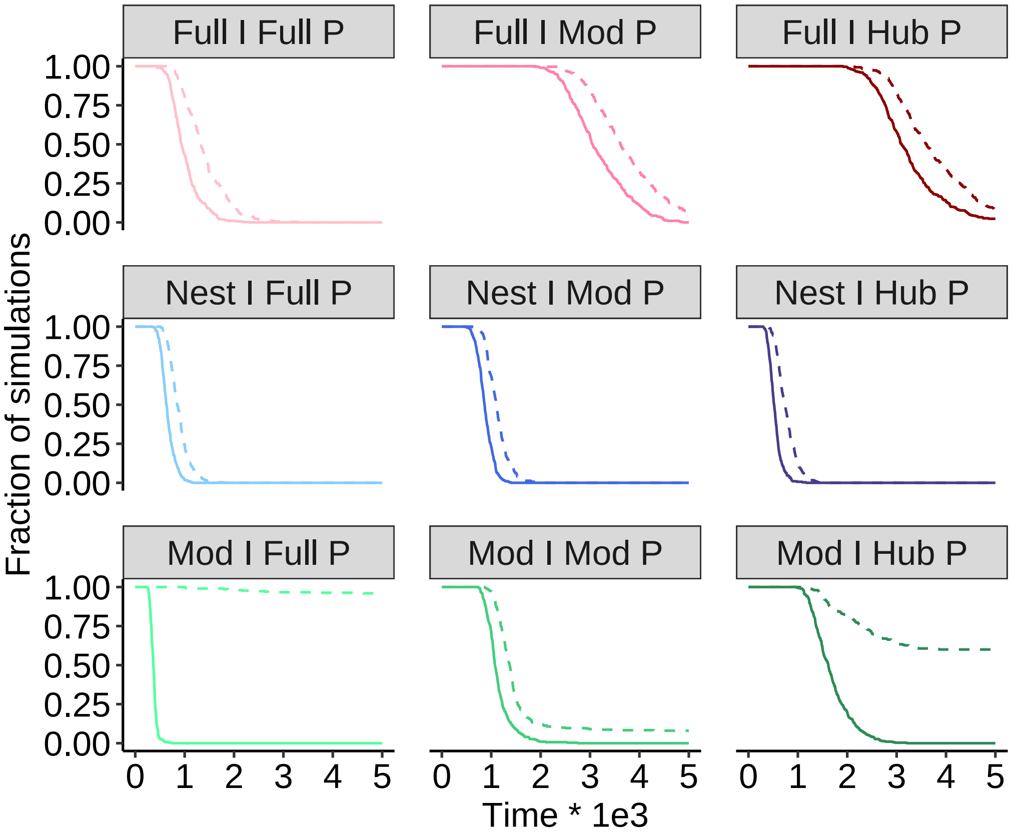


**Fig Q.** Dynamics of microbe coexistence of three populations (solid line) and two populations (dashed line) under density-dependent growth. The dynamics of coexistence probability (fraction of simulations) is calculated as the proportion of replicates with complete(3-population)/partial(2-population) coexistence out of total number of replicates across time. Time was only plotted to t = 5000 where most probabilities had dropped to zero or stabilized.

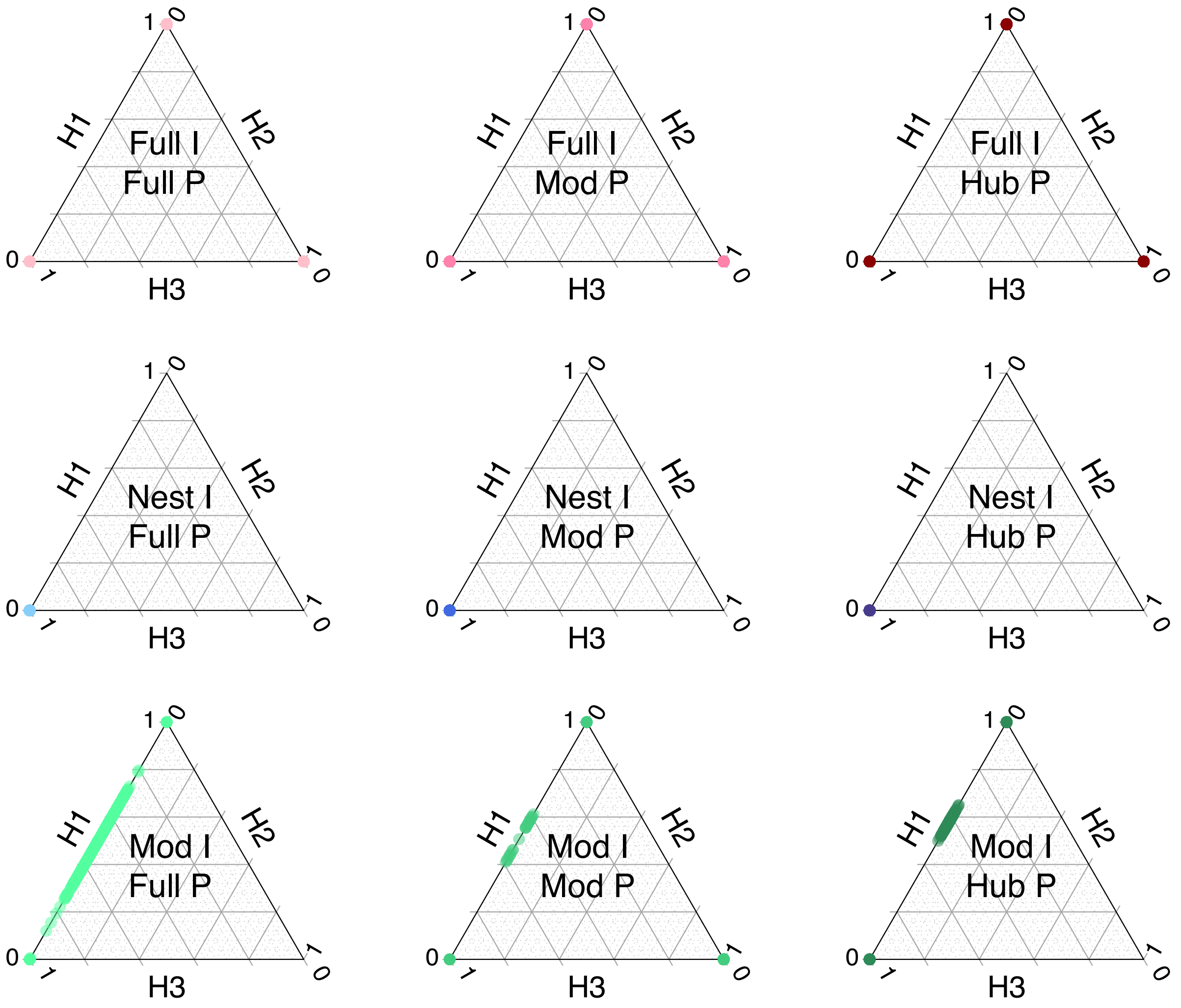


**Fig R.** Relative abundance of populations at the end of the simulations (t = 20000 hours) under density-dependent growth. Dots on the vertices represent no coexistence (i.e. only one population survived), while dots on the edges represent the coexistence of two populations.

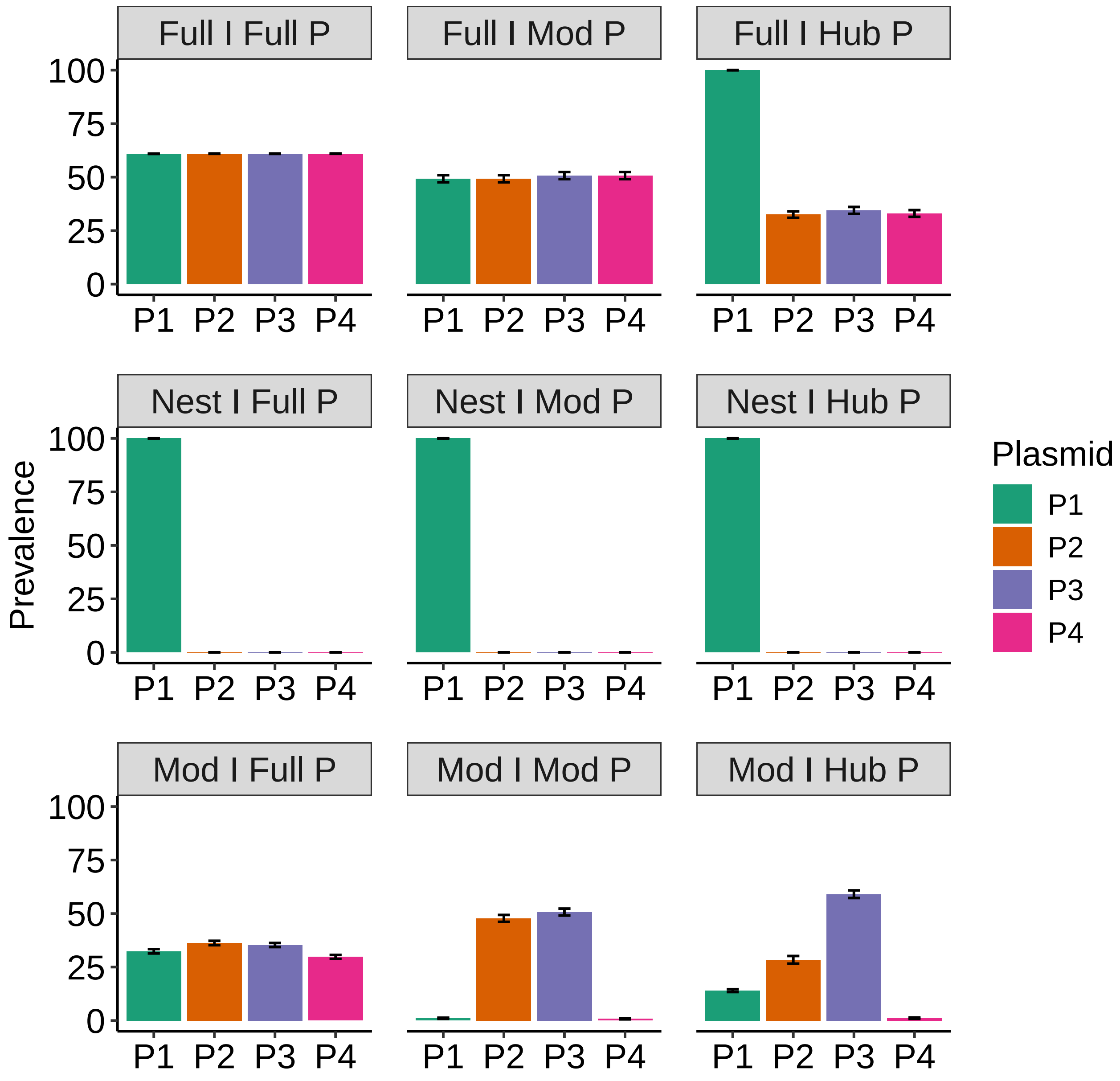


**Fig S.** Mean and SE of final plasmid prevalence across all replicates under density-dependent growth.

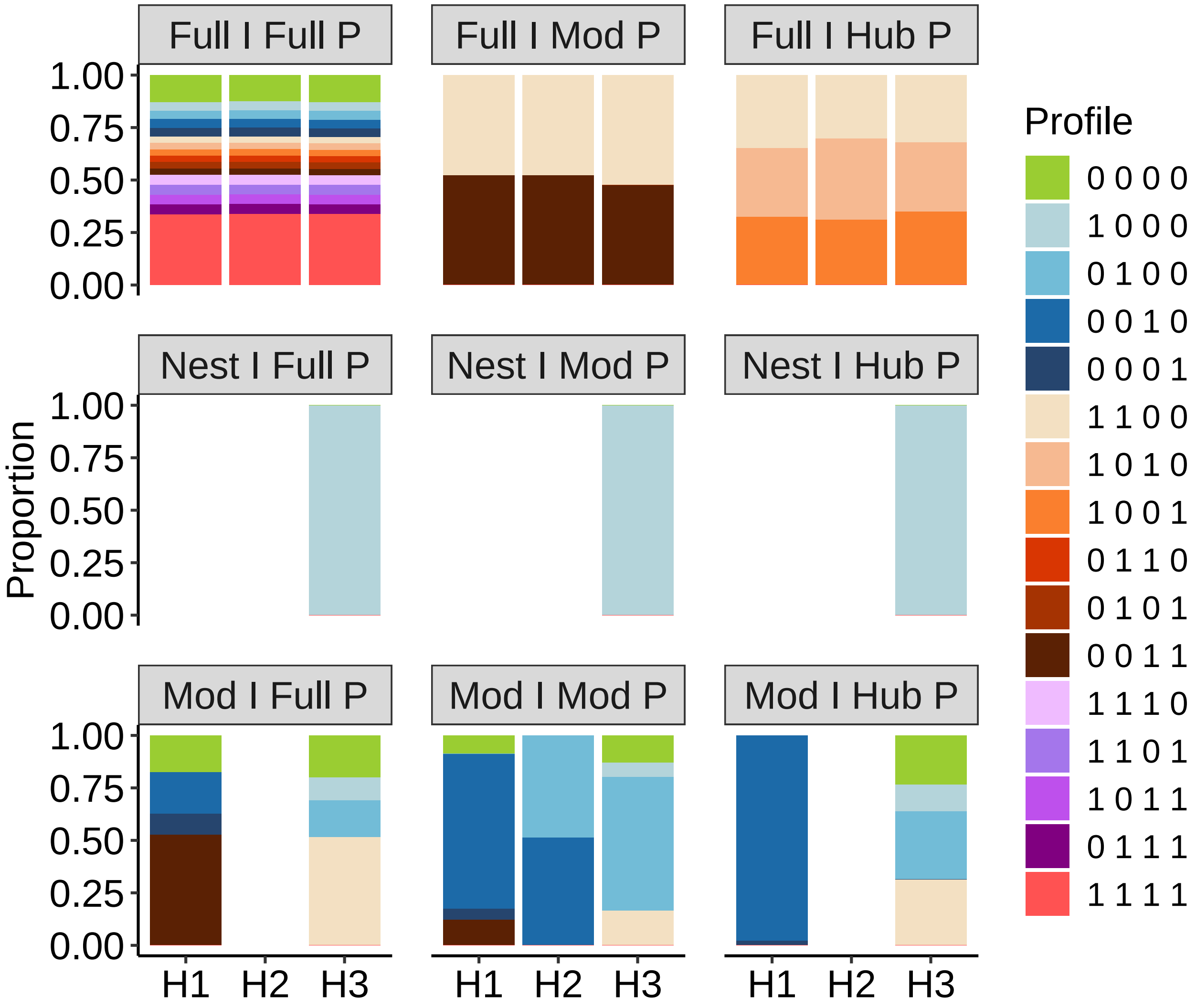


**Fig T.** Final host population composition under density-dependent growth, averaged across replicates in which that host population survived (only populations surviving in $> 5$ replicates were considered). Profiles represent the host subpopulations (e.g., the profile 1000 represents a subpopulation hosting only P1).

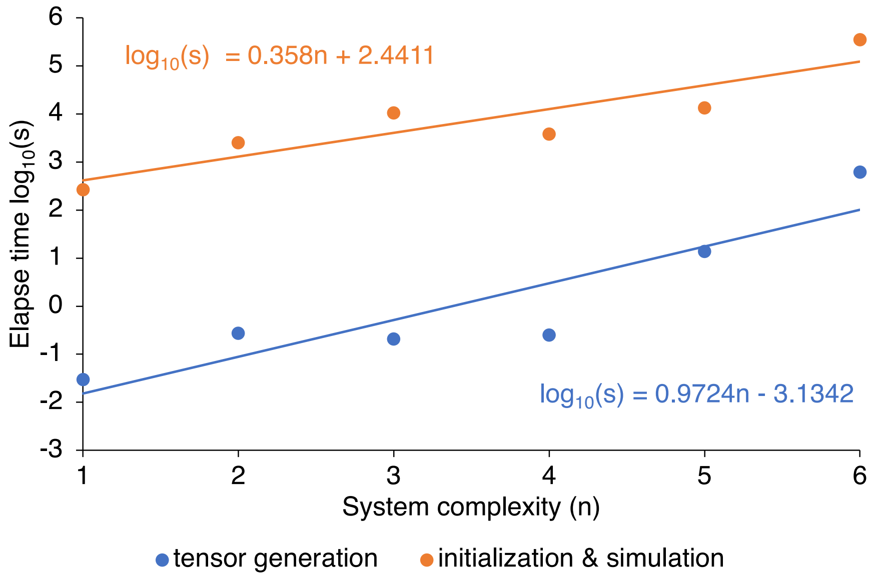


**Fig U.** Elapse time of tensor generation, initialization and simulation across system complexity $n$, i.e. the number of host and plasmid populations in the system.

# Tables A to I

**Table A.** Host-specific parameters. The net population growth rates were parameterized using the empirical data fit to the standard form of the logistic equation in the R package *growthcurver* [1], while the population growth rates and death rates were arbitrarily assigned to match the net population growth rate. The infection rates and competition coefficients were assigned in a relative magnitude in order to better match the empirical subpopulation and population dynamics. The segregation rates were fixed to an extremely low value so these can be negligible. The community-wise carrying capacity was arbitrarily assigned for computational efficiency. We later assumed an increase in the growth rate of the bridge host (H2).

| Host traits | H1 | H2 | H3 |
|---|---|---|---|
| growth rate $\eta_i$ | 0.1 | 0.15* | 0.2 |
| death rate $\mu_i$ | 0.01 | 0.01 | 0.01 |
| infection rate $\phi_{i,p}$ | 5e-9 | 5e-9 | 5e-8 |
| segregation rate $e_i$ | 1e-8 | 1e-8 | 1e-8 |
| competition coefficient on H1 $a_{1i}$ | 1 | 0.02 | 0.03 |
| competition coefficient on H2 $a_{2i}$ | 5e-3 | 1 | 0.03 |
| competition coefficient on H3 $a_{3i}$ | 5e-3 | 0.02 | 1 |
| perturbation impact $\epsilon_i$ | 0 | 0.0 | 0.0 |
| community-wise carrying capacity $K$ | 1e5 | 1e5 | 1e5 |

* Set to 0.1 under the assumption of no increase in the growth rate of H2.

**Table B.** Plasmid-specific parameters. The plasmid costs were assigned in a relative magnitude based on empirical monocultures of the infected bridge host population in order to better match the empirical subpopulation and population dynamics. The plasmid resistance to stress was arbitrarily assigned and fixed as these are irrelevant for systems without stress.

| Plasmid traits | P1 | P2 |
|---|---|---|
| plasmid cost $c_\alpha$ | 0.2 | 0.4 |
| plasmid resistance $\rho_\alpha$ | 0.7 | 0.7 |

**Table C.** Initial abundance of the plasmid-present system for a modular infection network **I**.

| Host $i$ | Plasmid profile $p$ | Abundance $H_{i,p}$ |
|---|---|---|
| 1 | [0,0] | 1650 |
| 1 | [1,0] | 1650 |
| 2 | [0,0] | 1650 |
| 2 | [1,0] | 550 |
| 2 | [0,1] | 550 |
| 2 | [1,1] | 550 |
| 3 | [0,0] | 1650 |
| 3 | [0,1] | 1650 |

**Table D.** Initial abundance of the plasmid-present system for a full infection network **I**.

| Host $i$ | Plasmid profile $p$ | Abundance $H_{i,p}$ |
|---|---|---|
| 1 | [0,0] | 1650 |
| 1 | [1,0] | 550 |
| 1 | [0,1] | 550 |
| 1 | [1,1] | 550 |
| 2 | [0,0] | 1650 |
| 2 | [1,0] | 550 |
| 2 | [0,1] | 550 |
| 2 | [1,1] | 550 |
| 3 | [0,0] | 1650 |
| 3 | [1,0] | 550 |
| 3 | [0,1] | 550 |
| 3 | [1,1] | 550 |

**Table E.** Constants and parameters for the model.

| Variable | Definition | Type |
|---|---|---|
| $t$ | time | float |
| $t_{final}$ | time scale of the simulation | float (h) |
| $n_b$ | number of host populations in the system | integer |
| $n_p$ | number of plasmids in the system | integer |
| $i, j, k$ | host of the entity | integer |
| $p, q, r$ | plasmid profile of the entity | integer |
| $\alpha$ | plasmid $\alpha$ | integer |
| $H_{i,p}$ | entity and abundance of a host subpopulation | integer |
| $H_i$ | abundance of host $i$ | integer |
| $K$ | community-wise carrying capacity of host | float |
| $e_i$ | probability of segregation error of host $i$ | float |
| $\mu_i$ | per capita death rate of host $i$ | float |
| $\eta_i$ | per capita growth rate of host $i$ | float |
| $\phi_{i,p}$ | per capita infection rate of host subpopulation $H_{i,p}$ | float |
| $\gamma_{i,p}$ | infection coefficient of host subpopulation $H_{i,p}$ | float |
| $\rho_\alpha$ | resistance of plasmid $\alpha$ | float |
| $c_\alpha$ | cost of plasmid $\alpha$ on host growth | float |
| $\mathbf{A}$ | host competition matrix | matrix of floats |
| $\mathbf{H}$ | HGT matrix | matrix of floats |
| $\mathbf{I}$ | infection matrix | matrix of integers |
| $\mathbf{P}$ | plasmid compatibility matrix | matrix of integers |
| $\mathbf{\Gamma}$ | propensity tensor of infection | 3-D tensor of floats |

**Table F.** Variables for the model input.

| Condition | Content | Type (v(): vector of ()) |
|---|---|---|
| t_final | t at which simulation ends | float |
| t_output | the t interval after which state is recorded | float |
| rng_seed | seed for the simulation | null; float |
| n_seeds | the number of seed for the simulation | integer; 1 |
| n_bstrains | $n_b$ maximum number of host populations in the system | integer |
| n_pstrains | $n_p$ maximum number of plasmids in the system | integer |
| n_bsubstrains | number of subpopulations at initial state | integer |
| strain_id_for_each_substrain | host id $i$ of subpopulations | integer |
| n_ind_bsubstrains | abundance of subpopulations | integer |
| p_profile_bsubstrains | the plasmid profile $p$ of subpopulations | v(v(s) of binary element(s)) |
| growth_rate | host-specific growth rate $\eta_i$ | v(float) |
| death_rate | host-specific death rate $\mu_i$ | v(float) |
| carrying_capacity | $K$ | v(integer) |
| infection_rate | host-specific infection coefficient $\gamma_i$ | v(float) |
| segregation_error | host-specific rate of segregation error $e_i$ | v(float(s)) |
| perturbation_impact | host-specific degradation coefficient $k_i$ | v(float / 0) w/o perturbation |
| plasmid_resistance | plasmid-specific plasmid resistance $\rho_\alpha$ | v(float) |
| plasmid_cost | plasmid-specific plasmid cost $c_\alpha$ | v(float) |
| A | host competition matrix $\mathbf{A}$ | v(v(float)) |
| H | host HGT matrix $\mathbf{H}$ | v(v(integer)) |
| I | plasmid infection matrix $\mathbf{I}$ | v(v(float(s))) |
| P | plasmid compatibility matrix $\mathbf{P}$ | v(v(float)) |
| tensor_file | path to the JSON file of the propensity tensor | character |

**Table G.** Constants and parameters for the model performance test.

| Variable | Definition | Value |
|---|---|---|
| $t_{final}$ | time scale of the simulation | 500 |
| $n$ | number of host and plasmid populations in the system | 1:6 |
| $K$ | community-wise carrying capacity of host | 1e4:2.1e5 |
| $e_i$ | probability of segregation error of host $i$ | e-8 |
| $\mu_i$ | per capita death rate of host $i$ | 0.015 |
| $\eta_i$ | per capita growth rate of host $i$ | 1.0 |
| $\gamma_{i,p}$ | infection coefficient of host subpopulation $H_{i,p}$ | 1e-4 |
| $\rho_\alpha$ | resistance of plasmid $\alpha$ | 1.0 |
| $c_\alpha$ | cost of plasmid $\alpha$ on host growth | 0.02 |
| $\mathbf{A}$ | host competition matrix | $n \times n$ matrix of 1s |
| $\mathbf{H}$ | HGT matrix | $n \times n$ matrix of 1s |
| $\mathbf{I}$ | infection matrix | $n \times n$ matrix of 1s |
| $\mathbf{P}$ | plasmid compatibility matrix | $n \times n$ matrix of 1s and off-diagonal 0s |

**Table H.** Quantified modularity (Q) and nestedness (NODF) of the designed bipartite infection networks **I**. Q (between 0 and 1 for natural ecological networks) is computed following Barber (2007) [2], while NODF (between 1 and 100) is computed following Almeida-Neto et al. (2008) [3].

| Network | Q | NODF |
|---|---|---|
| Full **I** | 0.0 | 0 |
| Nested **I** | $\approx 0.1$ | 100 |
| Modular **I** | $\approx 0.5$ | 0 |

**Table I.** Quantified Modularity (Qmax) and degree heterogeneity (H) of the designed unipartite plasmid compatibility networks **P**. Qmax (between 0 and 1 for natural ecological networks) is computed following Newman and Girvan (2004) [4], while H (between 0 and infinity; with 0.33 for a small network of four nodes indicating a star-like hub structure) is computed following Estrada (2010) [5].

| Network | Qmax | H |
|---|---|---|
| Full **P** | 0.0 | 0.0 |
| Modular **P** | 0.5 | 0.0 |
| Hub **P** | 0.0 | $\approx 0.33$ |

# References


1. Sprouffske K, Wagner A. Growthcurver: an R package for obtaining interpretable metrics from microbial growth curves. BMC Bioinformatics. 2016;17(1):172. doi:10.1186/s12859-016-1016-7.

2. Barber MJ. Modularity and community detection in bipartite networks. Phys Rev E Stat Nonlin Soft Matter Phys. 2007;76(6 Pt 2):066102. doi:10.1103/PhysRevE.76.066102.

3. Almeida-Neto M, Guimarães P, Guimarães PR Jr, Loyola RD, Ulrich W. A consistent metric for nestedness analysis in ecological systems: reconciling concept and measurement. Oikos. 2008;117(8):1227–1239. doi:10.1111/j.0030-1299.2008.16644.x.

4. Newman MEJ, Girvan M. Finding and evaluating community structure in networks. Phys Rev E Stat Nonlin Soft Matter Phys. 2004;69(2 Pt 2):026113. doi:10.1103/PhysRevE.69.026113.

5. Estrada E. Quantifying network heterogeneity. Phys Rev E Stat Nonlin Soft Matter Phys. 2010;82(6 Pt 2):066102. doi:10.1103/PhysRevE.82.066102.
